# Molecular modelling and site-directed mutagenesis provide insight into saccharide pyruvylation by the *Paenibacillus alvei* CsaB enzyme

**DOI:** 10.1038/s41598-023-40072-1

**Published:** 2023-08-17

**Authors:** Cordula Stefanović, Fiona F. Hager-Mair, Erik Breslmayr, Arturo López-Guzmán, Charlie Lim, Markus Blaukopf, Paul Kosma, Chris Oostenbrink, Roland Ludwig, Christina Schäffer

**Affiliations:** 1 NanoGlycobiology Research Group, Department of Chemistry, Institute of Biochemistry, Universität für Bodenkultur Wien, Muthgasse 18, 1190 Vienna, Austria; 2 Department of Bionanosciences, Institute of Biologically Inspired Materials, Universität für Bodenkultur Wien, Muthgasse 11, 1190 Vienna, Austria; 3 Department of Food Science and Technology, Biocatalysis and Biosensing Laboratory, Universität für Bodenkultur Wien, Muthgasse 11, 1190 Vienna, Austria; 4 Department of Material Sciences and Process Engineering, Institute for Molecular Modelling and Simulation, Universität für Bodenkultur Wien, Muthgasse 18, 1190 Vienna, Austria; 5 Department of Chemistry, Institute of Organic Chemistry, Universität für Bodenkultur Wien, Muthgasse 18, 1190 Vienna, Austria; 6 Present Address: Covirabio GmbH, Brehmstrasse 14a, 1110 Vienna, Austria

**Keywords:** Biochemistry, Chemical biology, Computational biology and bioinformatics, Microbiology, Structural biology

## Abstract

Pyruvylation is a biologically versatile but mechanistically unexplored saccharide modification. 4,6-Ketal pyruvylated *N*-acetylmannosamine within bacterial secondary cell wall polymers serves as a cell wall anchoring epitope for proteins possessing a terminal S-layer homology domain trimer. The pyruvyltransferase CsaB from *Paenibacillus alvei* served as a model to investigate the structural basis of the pyruvyltransfer reaction by a combination of molecular modelling and site-directed mutagenesis together with an enzyme assay using phosphoenolpyruvate (PEP; donor) and synthetic β-D-ManNAc-(1 → 4)-α-D-GlcNAc-diphosphoryl-11-phenoxyundecyl (acceptor). CsaB protein structure modelling was done using Phyre2 and I-Tasser based on the partial crystal structure of the *Schizosaccharomyces pombe* pyruvyltransferase Pvg1p and by AlphaFold. The models informed the construction of twelve CsaB mutants targeted at plausible PEP and acceptor binding sites and *K*_*M*_ and *k*_cat_ values were determined to evaluate the mutants, indicating the importance of a loop region for catalysis. R148, H308 and K328 were found to be critical to PEP binding and insight into acceptor binding was obtained from an analysis of Y14 and F16 mutants, confirming the modelled binding sites and interactions predicted using Molecular Operating Environment. These data lay the basis for future mechanistic studies of saccharide pyruvylation as a novel target for interference with bacterial cell wall assembly.

## Introduction

Molecular insight into the mechanisms of saccharide modifying enzymes is at the heart of modern glycoscience and provides the basis for the elucidation of structure–activity relationships and therapeutic intervention. Ketalpyruvyltransferases belong to a little investigated class of saccharide modifying enzymes^1^ that utilize 2-phosphoenolpyruvate (PEP) as a donor to form a pyruvate (Pyr) ketal bridging two hydroxyl groups of diverse monosaccharide residues thereby generating a ring structure which is most frequently placed across the 2,3-, 4,6-, or 3,4-positions^2^. Ketalpyruvylation can be found on diverse glycoconjugates in bacteria, yeasts and algae to which it imparts a net negative charge and mediates diverse pivotal biological functions, such as cell–cell interaction, influencing, *e.g.*, pathogenic adhesion, transmembrane trafficking, immunogenicity, and cell wall assembly (reviewed in Hager et al.^1^).

Ketalpyruvylation was studied in the fission yeast *Schizosaccharomyces pombe*, where the pyruvyltransferase Pvg1p (UniProt Q9UT27; 401 amino acid residues) equips a terminal β-galactose residue (β-Gal) of cell surface-located, complex eukaryotic *N*-glycans with a 4,6-*O*-[(R)-(1-carboxyethylidene)] modification (4,6-Pyr)^3,4^. Pvg1p is a Golgi-resident enzyme and relies on the Pet1p and Pet2p transporters for the supply of PEP from the cytoplasm^5^. Evidence of Pvg1p activity was obtained by NMR analysis of an HPLC-purified enzyme reaction product using recombinant Pvg1p together with PEP as substrate and *p*-nitrophenyl-β-Gal (*p*NP-β-Gal) as acceptor^3^; other suitable acceptors in the Pvg1p reaction were free lactose (Lac; Gal-β1,4-Glc) intended to mimic the terminal β-Gal residue on the native *N*-glycans (albeit, these terminate with a Gal-β1,3-Gal-α1-disaccharide) and *p*NP-attached Gal-β1,4-GlcNAc (LacNAc) representing a frequent disaccharide terminus of human-type complex *N*-glycans^6^. A truncated version of Pvg1p (corresponding to the D32-F401 portion of the enzyme devoid of the N-terminal part including a predicted membrane spanning segment) was produced in *E. coli* and the crystal structure of the apoenzyme was determined in the presence of Zn^2+^ at a resolution of 2.46 Å (pdb 5ax7)^7^. In the available crystal structure, protein stretches spanning amino acids L33-D52 and R264-D291 are disordered and the stretch from D268 through T284 is missing. It was revealed that two Pvg1p molecules form an asymmetric unit with a two-fold axis (homodimer) and approximately 928 Å^2^ of the surface area is buried at the dimer interface. The orientation of the N-termini of both protomers on one side of the dimer is consistent with a type II membrane-bound protein where the protomers would be anchored to the Golgi membrane via their N-terminal transmembrane domains^7^. Since Pvg1p was not amenable to co-crystallization with neither PEP nor the tested acceptor substrates, current knowledge of the enzymatic mechanism is derived from a substrate-bound model of Pvg1p with PEP together with the non-native acceptors Lac and LacNAc, respectively, in combination with very limited mutational analysis (targeted at amino acids D106 and H168, respectively)^7^. According to the Pvg1p-PEP-Lac model, residues R217 and R337 of Pvg1p interact with the negatively charged PEP substrate, consistent with their location in a positively charged cleft situated between the N- and C-terminal halves of Pvg1p; D106 which is situated within hydrogen bond-forming distance of the O6 oxygen of the Gal residue of Lac is involved in acceptor binding, as was concluded from the inactivity of a D106A variant. For binding of LacNAc, H168 is a steric hindrance supported by superior binding of the acceptor to a H168A and a H168C Pvg1p variant, respectively. However, since LacNAc is an artificial acceptor, this finding is only of limited relevance for the elucidation of the catalytic mechanism of the enzyme. Other active-site amino acids predicted from the modeled enzyme–substrate complexes include H101, N103 and Y165 of Pvg1p^7^. The authors of that study concluded that dimerization of Pvg1p is not required for substrate binding and catalysis, since the predicted active site localizes far away from the dimer interface^7^.

The CsaB enzyme (Uniprot K4ZGN3; 396 amino acids) of the Gram-positive bacterium *Paenibacillus alvei* is another ketalpyruvyltransferase, which has so far been studied from a biochemical perspective^8,9^. It catalyzes the modification of the *N*-acetylmannosamine (ManNAc) residue within [→ 4)-β-D-GlcNAc-(1 → 3)-β-D-ManNAc-(1 →] disaccharide repeats which build up the bacterium’s secondary cell wall polymer (SCWP)^10^. 4,6-Ketalpyruvyation of ManNAc is essential for the SCWP to serve as a cell wall ligand for the bacterium’s cell surface (S-) layer (SpaA) glycoprotein array^11^. Of note, 4–6-pyruvylated β-D-ManNAc of SCWPs is a universal epitope for the interaction with S-layer homology(SLH) domain-containing cell surface proteins (precisely, with the N-terminal SLH domain trimer), such as the cell surface proteins SpaA and SlhA of *P. alvei*^12^ as well as the S-layer and other cell surface proteins of the pathogen *Bacillus anthracis*^13,14^, among others. Thus, the 4,6-Pyr-ManNAc-SLH domain trimer interaction is decisive for the cell wall architecture and integrity of several Gram-positive bacteria^12,13,15,16^. *Paenibacillus alvei* CsaB requires PEP as a donor substrate and the presence of a lipophilic appendage on the acceptor substrate for in vitro activity, as was shown with a synthetic, tailor-made β-D-ManNAc-(1 → 4)-α-D-GlcNAc-diphosphoryl-11-phenoxyundecyl (ManNAc-GlcNAc-PP-UndPh) acceptor precursor analogue, where the UndPh-P portion mimics the endogenous lipid-carrier undecaprenylphosphate^8^. Consequently, it is conceivable to assume that *P. alvei* CsaB is active at the biosynthetic stage of a cytoplasmic lipid-linked SCWP disaccharide repeat precursor prior to translocation of the pyruvylated SCWP precursor across the cytoplasmic membrane and polymerization of the repeats into the mature SCWP^17^. In contrast, the CsaB ortholog of *B. anthracis* (Uniprot Q9L471; 367 amino acid residues)^18,19^, where the pyruvate modification is present exclusively at the terminal β-D-ManNAc residue of the terminal SCWP trisaccharide and not on the ManNAc residues within the SCWP repeats, is assumed to be active at the exterior face of the cytoplasmic membrane^20^, in analogy to the *B. anthracis* acetyltransferase which is active on the same SCWP trisaccharide^21^. However, there is no experimental evidence available to support this assumption.

Analysis of the taxonomic distribution of CsaB-like and Pvg1p-like pyruvyltransferases in sequence similarity networks indicated that the former enzymes mainly occur in the bacterial phyla of *Firmicutes* and *Cyanobacteria*, while the latter, apart from fungi (*Ascomycota*), in those of *Proteobacteria* and, to a lesser extent, *Firmicutes*^1^. Whether this distribution has implications for the catalytic mechanism of pyruvyltransferases is unknown.

In the present study, we used the well-expressed, full-size, soluble *P. alvei* CsaB enzyme in order to gain insight into the structural basis of the pyruvyltransfer reaction and the enzyme’s substrate specificity. Computer modelling based on the partial *S. pombe* Pvg1p crystal structure using Phyre2 and I-Tasser as well as ab-initio protein structure modelling by AlphaFold together with small molecule docking using Molecular Operating Environment (MOE) (v2019) were performed to reveal putative catalytic amino acid residues for mutational studies. The activity of twelve *P. alvei* CsaB variants was screened using our recently developed quantitative colorimetric ketalpyruvyltransferase assay^9^. We report on amino acid residues of CsaB important for the pyruvyl transfer mechanism onto β-D-ManNAc based on the determined kinetic constants including *K*_M_ and *k*_cat_ values for the donor substrate (PEP) and the acceptor for all generated enzyme variants and reveal a loop region that is likely important to the activity of saccharide::ketalpyruvyltransferases.

## Results

### Modelling of CsaB

**Figure 1:**
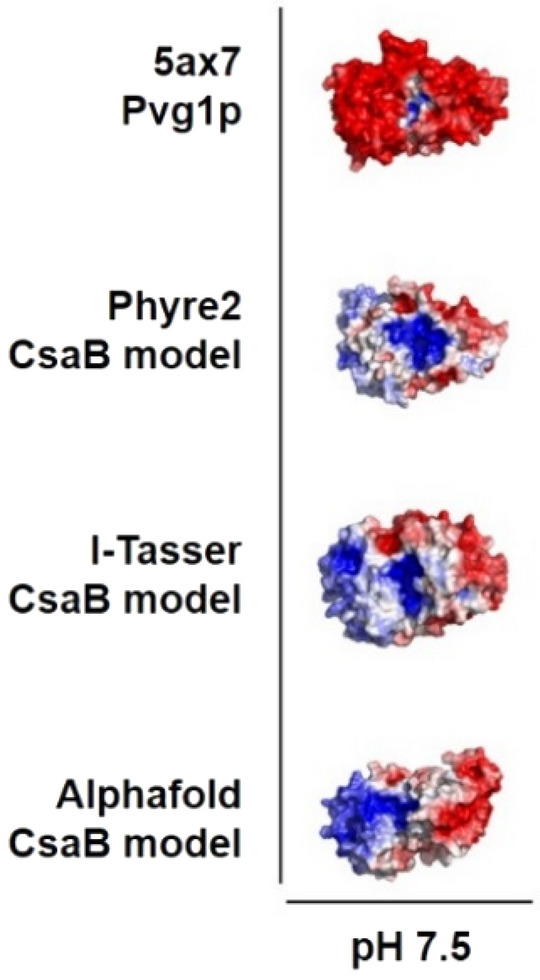
Surface electrostatics of Pvg1p from *S. pombe* (5ax7) in comparison to models of CsaB from *P. alvei* at pH 7.5. Surface areas with a positive electrostatic potential (+5 *RT/e*) are colored in blue, surface areas with a negative electrostatic potential (− 5 *RT/e*) in red. Surface electrostatics were calculated using pHmap (v1.2)^22^, which automatizes the usage of ABPS (v3)^23^, pdb2pqr (v2.1.1) (https://www.poissonboltzmann.org/) and PyMol (v2.4).

**Figure 2:**
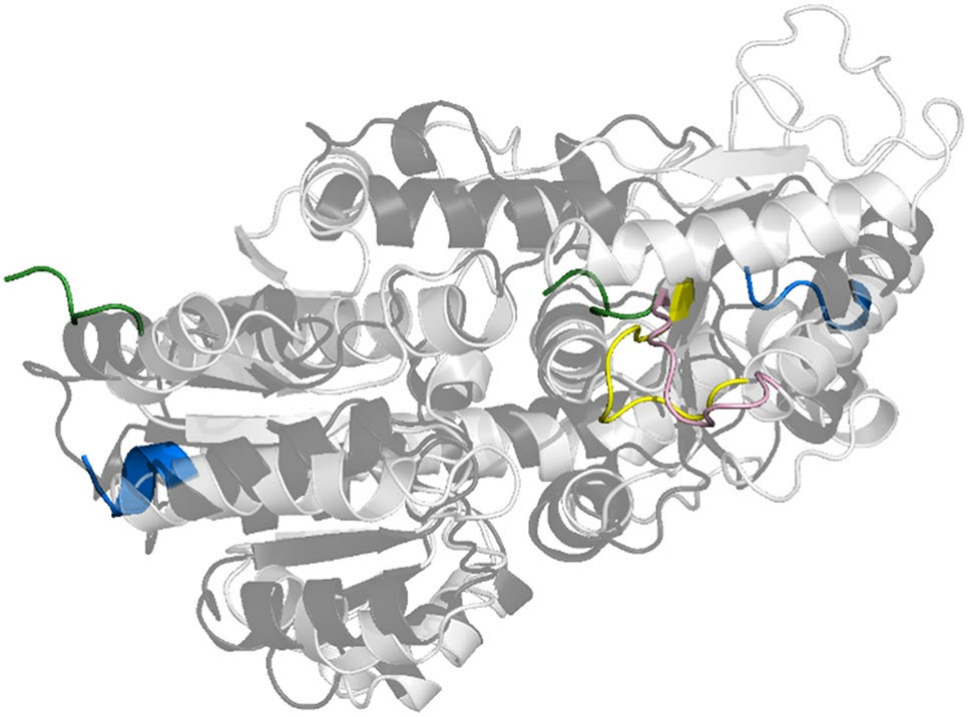
Overlay of the AlphaFold model of the CsaB enzyme (light grey) and the Pvg1p enzyme (black) with the loop region (“loop 1”) shown in rose (CsaB) and yellow (Pvg1p), respectively. The N-terminus of the enzymes is colored in green and the C-terminus in blue, showing the five terminal amino acids, each. CsaB and Pvg1p protein structures were visualized by PyMoL (Open Source Version 2.4; https://github.com/schrodinger/pymol-open-source).

The CsaB enzyme (Uniprot K4ZGN3; 396 amino acids) was homology-modeled by use of Phyre2 and I-Tasser as well as by AlphaFold. The first two of these programs based their modeling approach onto the only so far crystallized pyruvyltransferase, Pvg1p (pdb 5ax7^7^), which shows a low sequence identity with CsaB of ~ 20% but, still, is the template with the highest score. For comparison of the different models and as a prerequisite for small molecule docking studies of the donor and the acceptor substrate, surface electrostatics of CsaB between a pH 4 and 11 were calculated. These showed significant differences between the surface charges in CsaB compared to Pvg1p (5ax7), but similarities with regards to the presence of a positively charged cleft situated between the N- and C-terminal halves of the enzymes (Supplementary Fig. S1) representing the potential PEP binding site^7^. Figure 1 shows the surface electrostatics of the enzymes at pH 7.5, which is the previously determined pH optimum for CsaB activity^9^ and the pH value at which the truncated version of Pvg1p was crystallized^7^. Note that the CsaB models differ in the exact location of a further positively charged patch at the left side of these figures. In the AlphaFold model, it is connected to the active site cleft, while in the other two models it appears more shifted towards the left, respectively the back of the protein structure (see Supplementary Fig. S1). This is due to smaller changes in the loops connecting the helices to the sheets (Fig. 2).

The Phyre2, I-Tasser and AlphaFold models of the CsaB structure were of overall high confidence. The Phyre2 model only covered the resolved parts of the template, while I-Tasser predicted some parts as random coil. AlphaFold predicted the entire sequence, with an average pLDDT value of 90.76 over 396 residues. Two loops at the back of the protein (far removed from the active site) show pLDDT values that drop down to values of about 40. Of notable interest, however, is the flexible region spanning amino acids S205 through R213 (named “loop 1”). Phyre2 predicted for this region a random coil structure, while I-Tasser and AlphaFold predicted a loop attached to the CsaB structure, AlphaFold with an average pLDDT value of 94.93, indicating high confidence. The AlphaFold model of CsaB showed the highest similarity to the 5ax7 template and represented a good predicted model of surface exposed loops (no random coils), which could not be modelled by Phyre2 (based on the 5ax7 template with a confidence of 100%) and only moderately with I-Tasser (where 5ax7 was best ranked). Hence, AlphaFold delivered the most reliable model of the CsaB structure (which was confirmed by experimental data as detailed below). A search for structurally similar proteins was performed with this model on the protein databank (https://www.rcsb.org). This resulted in a list of 46 proteins. Four of these were characterized as glucose-1-phosphate adenyltransferase (3BRK, 6VR0, 5L6S, 5W6J), two as mannose-1-phosphate guanylyltransferase (7X8K, 7X8J). In addition, nine dehydrogenases, seven methyl transferases, and five glutamate receptors were found.

We also modelled the Pvg1p sequence with AlphaFold, revealing also in this enzyme a flexible region spanning amino acids L264-Q274 (Fig. 2). Notably, the stretch from D268 through T284 in Pvg1p including the predicted loop is missing in the X-ray structure of the *S. pombe* enzyme (5ax7). This sequence stretch, however, might be of importance for substrate binding, since it partially covers the active site in the previously modelled Pvg1p-PEP-Lac complex, using the MOE software^7^.

### Small molecule docking for selection of mutation sites in CsaB

**Figure 3:**
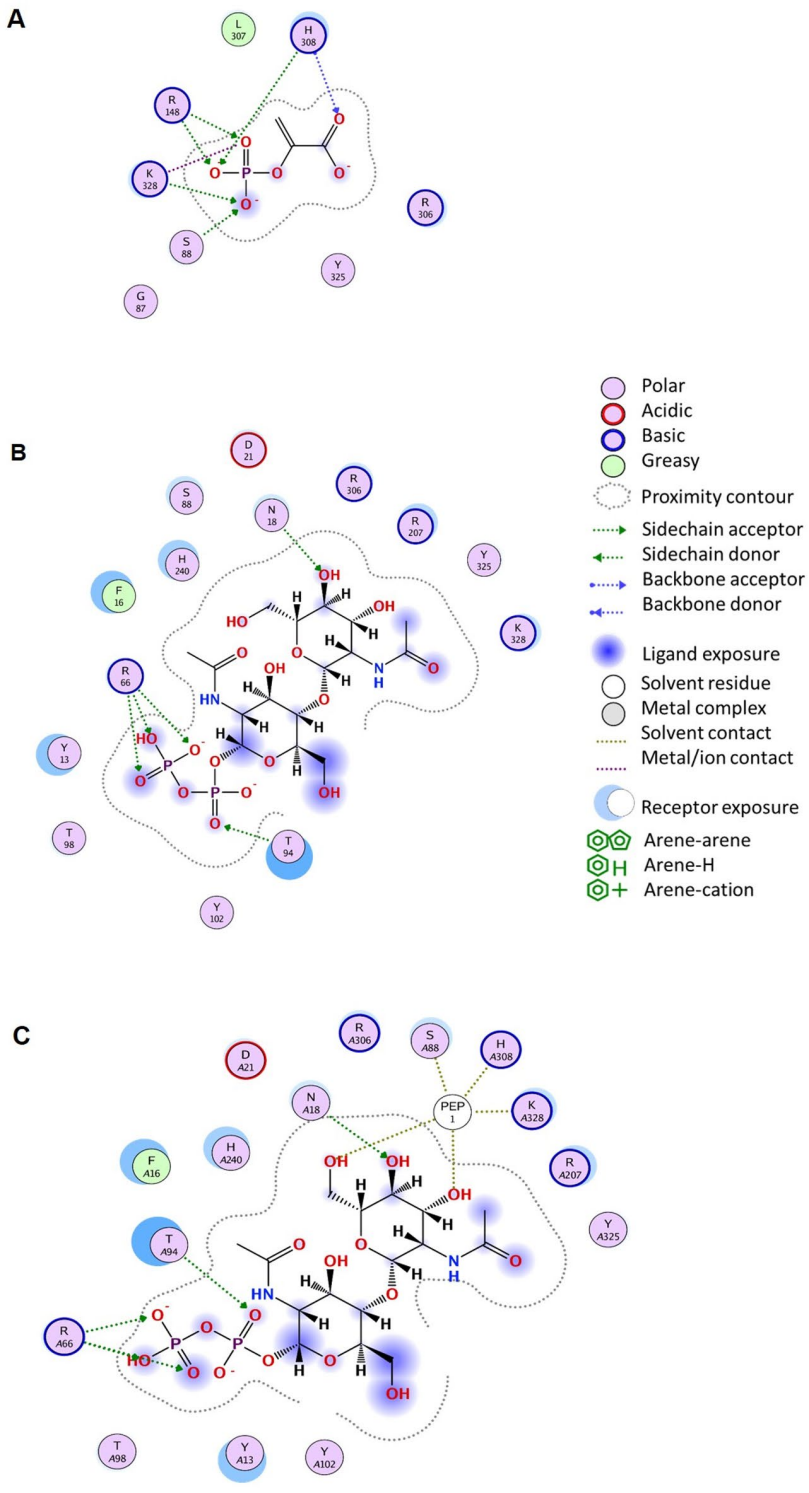
Ligand interaction diagrams for the PEP donor (**A**), the acceptor substrate (**B**) and the acceptor substrate in the presence of PEP (**C**), each docked to the binding site in the AlphaFold model of CsaB. Represented interacting residues of the CsaB are within a distance of 4 Å of either substrate. *A* before an amino acid position signifies docking of two substrates to the respective amino acid. Interaction diagrams were done in MOE (Molecular Operating Environment, v2019).

**Figure 4:**
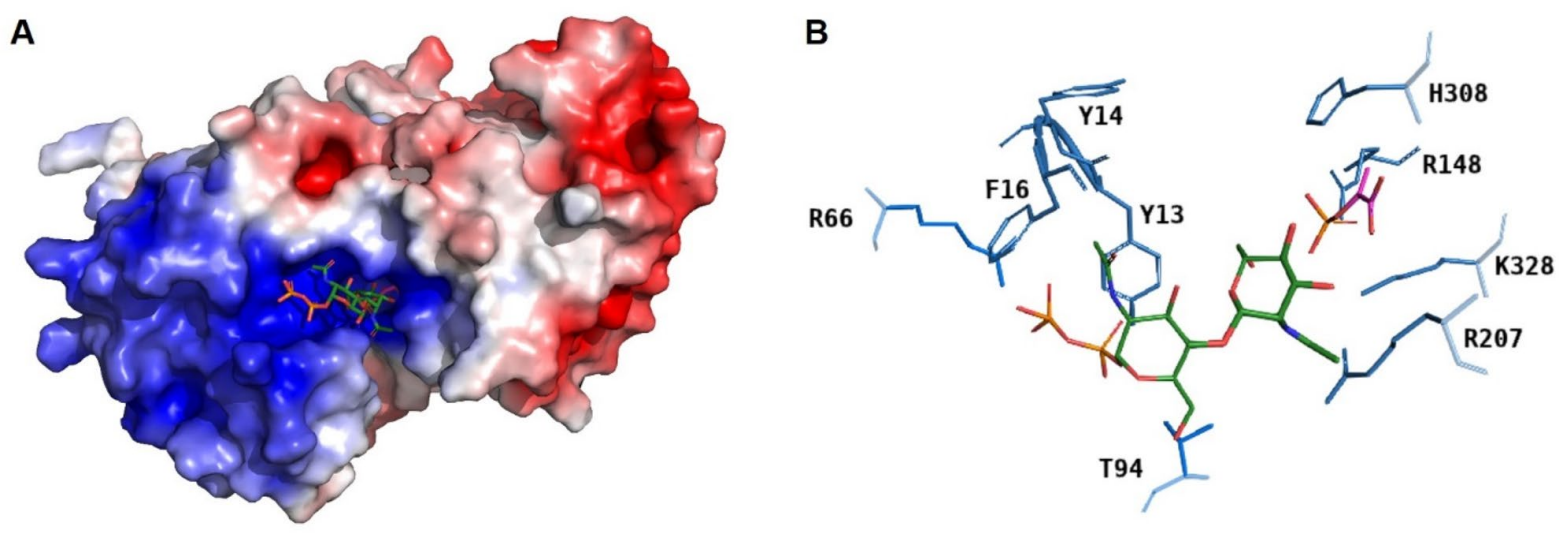
Surface electrostatics of CsaB at pH 7.5 calculated based on the AlphaFold model of the enzyme, with docked PEP (magenta) and acceptor substrate (green; phosphates in orange) (**A**). Zoomed-in view of the AlphaFold docking model of CsaB (blue) with PEP (magenta) and acceptor substrate (green; phosphates in orange) to show amino acid residues within 4-Å distance to the substrates (hydrogen bonds are hidden) (**B**). Surface electrostatics were calculated using pHmap (v1.2)^22^, which automatizes the usage of ABPS (v3)^23^, pdb2pqr (v2.1.1) (https://www.poissonboltzmann.org/) and PyMol (v2.4).

Based on the surface electrostatics (Fig. 1 and Supplementary Fig. S1) and the literature data about Pvg1p^7^, docking studies of CsaB with PEP and β-D-ManNAc-(1 → 4)-α-D-GlcNAc-PP which served as an acceptor mimic were done using MOE. In Fig. 3, the architecture of the active site in the AlphaFold model of CsaB for the PEP donor (Fig. 3A), the acceptor (Fig. 3B) and both substrates (Fig. 3C; compare with Fig. 4) is shown. Docking the donor to the modeled structure of CsaB resulted in a similar position of the PEP substrate compared to 5ax7 (Supplementary Fig. S2) and indicated the amino acid residues R148 (i.e., R217 in 5ax7), H308 (i.e., H339 in 5ax7), K328 (i.e., K361 in 5ax7) and R306 (i.e., h R337 in 5ax7) as well as S88 (no correspondence in 5ax7; instead, G159) as potential PEP binding sites. Contrary to the literature data for the yeast enzyme^7^, the PEP docking with neither CsaB (this study; compare with Fig. 3A) nor 5ax7 (this study, Supplementary Fig. S3) revealed clear interactions with leucine residue (i.e., L338 of 5ax7). Instead, based on the docking of PEP to both enzyme models, K328 of CsaB (corresponding to K361 in 5ax7) is predicted to be involved in PEP binding (Fig. 3A). Concerning the situation at the acceptor binding site, the ligand interaction diagram of CsaB with the acceptor indicated the residues Y13, F16, R66 and T94 of CsaB to be involved in binding (Fig. 3B).

Considering the surface electrostatics of CsaB calculated at pH 7.5 based on the AlphaFold model in conjunction with docked PEP and docked acceptor (donor and acceptor substrate were not present during surface charge calculation), it is clearly visible that the docked position of the acceptor points with the diphosphates to the more positive region of the enzyme (Fig. 4A). This gives further confidence to the selected docking position and supports the involvement of the residues F16, Y13, Y14, R66 and T94 in binding (Fig. 4B) as was also indicated in the ligand interaction diagram (Fig. 3B). Of note, regarding the acceptor binding site, 5ax7 is not representative for the study of CsaB, because these enzyme orthologs require structurally different acceptor substrates, i.e., Gal or Lac for Pvg1p^3^ versus ManNAc-GlcNAc-PP for CsaB^8^.

**Figure 5:**
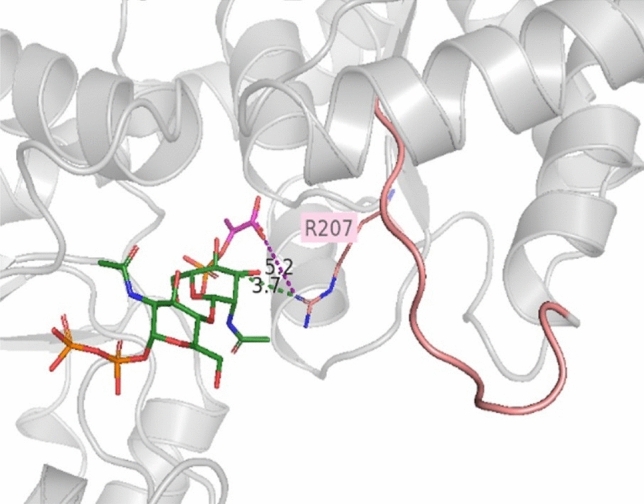
Enlarged view of “loop 1” (rose) in CsaB modelled by AlphaFold with docked substrates. The conserved arginine residue R207 of the enzyme and its distance to PEP (in magenta; distance shown as dotted line in magenta) and the acceptor (green with phosphates in orange; distance shown as dotted line in green) is indicated. The CsaB protein structure with docked substrates was visualized by PyMoL (Open Source Version 2.4; https://github.com/schrodinger/pymol-open-source).

To investigate a potential role of “loop 1” in the CsaB pyruvyltransferase reaction, a conserved arginine residue, i.e., R207 within CsaB (corresponding to R266 in the 5ax7 structure) was mutated to aspartate. This fully deactivated the enzyme and demonstrated the importance of the loop for catalysis (compare with Table 1). Given the position of R207 in the CsaB AlphaFold model (Fig. 4B), we reasoned that this amino acid might be involved in substrate binding, implicating that “loop 1” is located closely to the active site. In the AlphaFold model of CsaB, the closest atom of PEP to R207 is at 5.2-Å distance, the closest atom of the acceptor to R207 is at 3.7-Å distance (Fig. 5). For comparison, “loop 1” modelled by Phyre2 is further away from the active site of CsaB compared to the situation in the AlphaFold model (Supplementary Fig. S4).

**Table 1:**
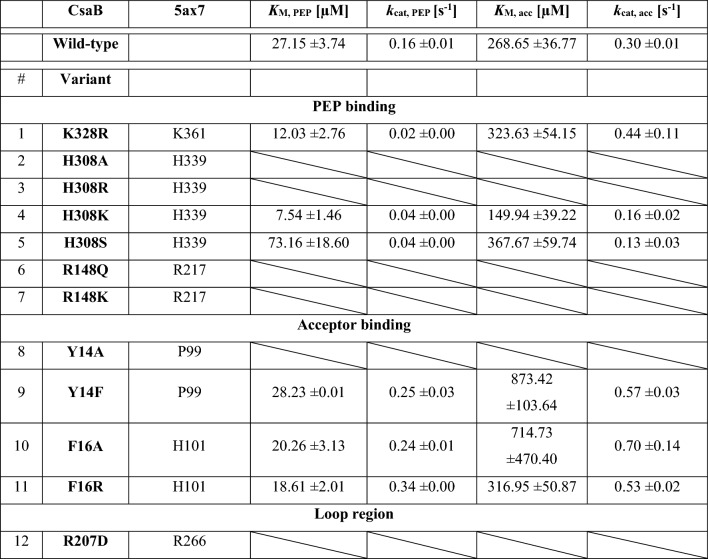
Kinetic characterization of CsaB variants in comparison to the CsaB wild-type enzyme. Mutation sites are conserved between CsaB and Pvg1p (5ax7).

Inactivity of enzymes is represented by a diagonal split.

### Assessment of structural integrity of CsaB wild-type and variants

Wild-type CsaB and the CsaB variants Y14A, Y14F, F16A, F16R, R148K, R148Q R207D, H308A, H308K, H308S, H308R, and K328R were obtained in good yield in *E. coli* which was used as a host for recombinant protein production (i.e., 0.36, 0.34, 0.42, 0.40, 0.38, 0.40, 0.40, 0.32, 0.38 mg/ml of *E. coli* culture) and good purity after Ni–NTA purification according to SDS-PAGE evidence (Supplementary Fig. S5). Of note, the amino acids S88 and T94, which were predicted to be involved in PEP and acceptor binding, respectively, (compare with Fig. 3) could not be mutated and, thus, not investigated experimentally.

All recombinant CsaB variants except for the Y14F, H308S and K328R variants exhibited a wild-type-like overall secondary structure composition according to the curve shape in the far-UV as demonstrated by electronic circular dichroism (ECD) spectroscopy (Supplementary Fig. S6). The Y14F, H308S and K328R variants were similar among each other, but differed slightly from the wild-type curve shape (Supplementary Fig. S7). However, according to the calculation after ECD analysis using Context Dependent Neural Networks (CDNN), all variants have a very similar secondary structure content comparable to the wild-type protein, containing 15–25% α-helices, 18–20% β-sheets and 41–55% random coils (Supplementary Fig. S8).

AlphaFold models of the three variants (i.e., Y14F, H308S and K328R) which showed slightly different secondary structure integrity were generated. After global confidence ranking, the models were superimposed (Supplementary Fig. S9), showing uncertainty and structural differences in another predicted loop region between residues 180–193 (named “loop 2”). To further assign the differences in the structures of Y14F, H308S and K328R compared to the CsaB wild-type protein, the DSSP plugin tool in PyMol was used (Supplementary Table S1). The data supports the CD spectroscopy results and the AlphaFold models, as small changes in the secondary structure could be determined, however not at the mutation sites, but in the adjacent “loop 2”.

### Activity testing and kinetic characterization of CsaB variants

**Figure 6:**
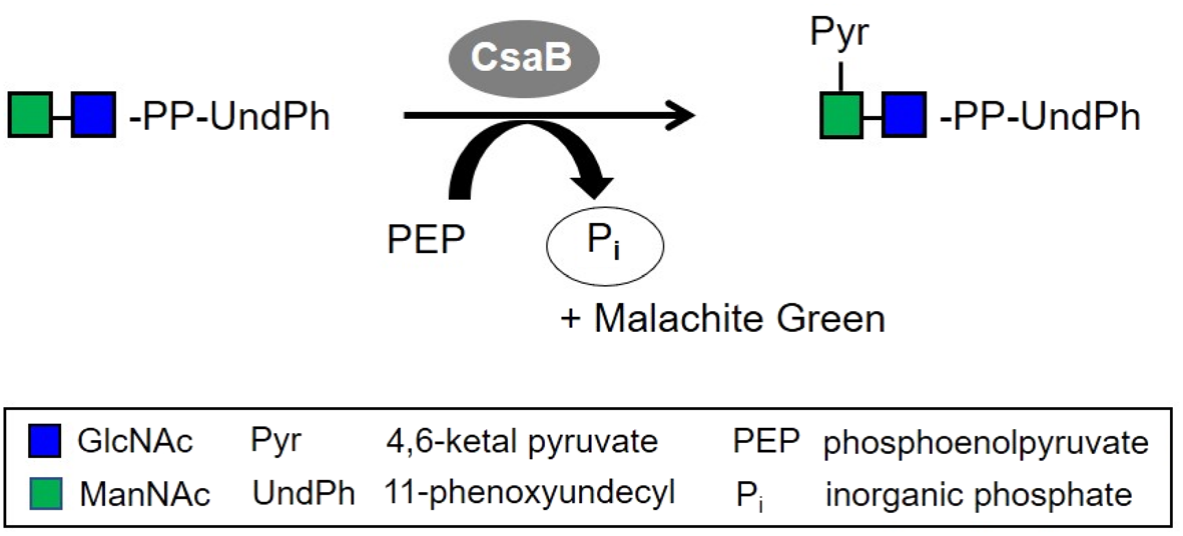
CsaB activity assay^9^. Colorimetric quantification of the green complex formed between Malachite Green, molybdate and free phosphate released from PEP upon enzymatic pyruvate transfer to the ManNAc-GlcNAc-PP-UndPh acceptor by measuring the absorbance at 620 nm. Monosaccharide symbols are shown according to the Symbol Nomenclature for Glycans (SNFG)^24^.

Six of the twelve constructed CsaB variants proved to be active in a standard CsaB activity assay^9^ (Fig. 6); these were K328R, H308K, H308S, Y14F, F16A and F16R (Table 1).

**Figure 7:**
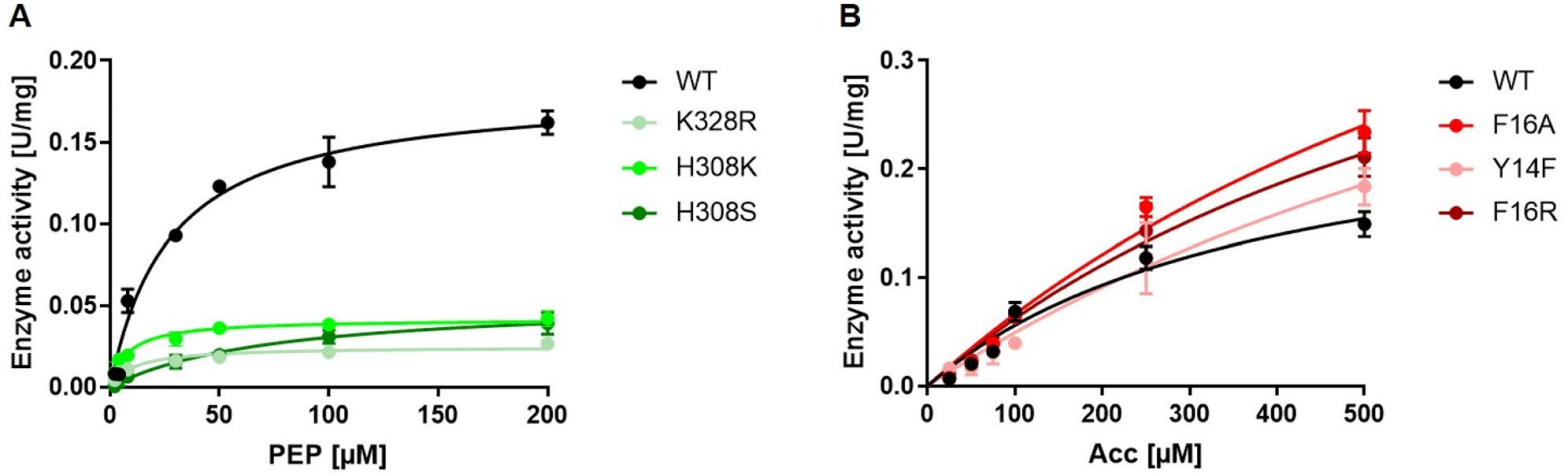
Plots of CsaB enzyme activity versus substrate concentration revealing *K*_M_ and *k*_cat_ values (also see Table 1). (**A**) Direct Michaelis–Menten plot for varying PEP concentrations and (**B**) for varying acceptor substrate concentrations. GraphPad Prism (version 9.1.2; GraphPad, San Diego, CA, USA) was used for statistical analysis, where *K*_M_ and *V*_max_ values were calculated by non-linear least-square regression to the direct Michaelis–Menten plot.

Subsequently, kinetic analysis of these variants was performed in comparison to the CsaB wild-type enzyme to determine *K*_M_ and *k*_cat_ values for the PEP donor substrate and the acceptor substrate. The values are reported as *K*_M, acc_ and *K*_M, PEP_ as well as *k*_cat, acc_ and *k*_cat, PEP_ and are based on the concentration-dependent activity plots for PEP (Fig. 7A) and acceptor substrate (Fig. 7B). For a summary of the kinetic characterization of the CsaB variants see Table 1 (A slight difference in the kinetic constants − *K*_M, acc_, *K*_M, PEP_ and *k*_cat, acc_ and *k*_cat, PEP_—compared to the recently published values for the CsaB wild-type enzyme^9^ is due to the use of a different batch of the acceptor substrate in the present study).

The K328R variant, which targeted the predicted catalytic site, reduced the *k*_cat_ approximately eightfold for PEP, but did not fully deactivate the enzyme. Thus, it is unlikely that K328 is a critical part of the catalytic site, but it might be more involved in PEP binding, since the *K*_M_ of PEP was lowered 2.2-fold. In comparison, the affinity of the acceptor substrate *K*_M, acc_ was little affected and increased slightly, as well as the *k*_cat, acc_. The decrease of *K*_M, PEP_ could arise from the introduced additional electrostatic interactions of which the arginine side chain is capable of compared to lysine, which might lead to a stickier environment for PEP in the active site.

The set of H308A/R/K/S and R148Q/K variants was generated to examine the predicted PEP-binding site. Two mutations at position 308 (H308A, H308R) fully deactivated the variant enzymes, demonstrating the importance of H308 as part of the PEP binding site. The alanine residue at this position is quite small and might lead to a reorientation of other close side chains as well as the formation of a water pocket, while the arginine residue supposedly is too big and results in a steric clash with PEP in the binding site. However, the two other mutations (H308K and H308S) did not fully deactivate the variant, suggesting that the other PEP binding residues can compensate a loss in the PEP binding capability. The lysine residue in the H308K variant can replace the histidine residue in the binding of the co-substrate; it shows a 3.6-fold increased affinity for PEP, however, it also reduces the *k*_cat, PEP_ by 3.8-fold, which might be a similar effect as for K328R. H308S reduces *k*_cat, PEP_ to a similar extent, but changes the *K*_M, PEP_ only moderately (2.8-fold) by not providing a positive counterpart for the negative charge from the phosphate group of PEP. Replacement of R148 by a hydrophilic, but uncharged amino acid (glutamine; R148Q) or a smaller charged amino acid (lysine; R148K) resulted in the full loss of enzymatic activity indicating a strong involvement of R148 in PEP binding or catalysis. The obtained kinetic constants for the variants at the three mutation sites K328, H308 and R148 strongly indicate that the PEP binding site is located at the position suggested by docking (Figs. 3A, 4).

A second set of variants, Y14A/F and F16A/R, was designed to investigate the acceptor binding site. Apart from Y14A, which gave no detectable activity, Y14F increased *K*_M_ and *k*_cat_ of the acceptor substrate, but not of PEP. Likewise, F16A/R increased the *K*_M_ value for the acceptor, but did not foster a change of the kinetic constants for PEP. These results are in agreement with the proposed docking pose and suggest that acceptor substrate binding occurs in the assigned pocket (Fig. 4). Interestingly, the exchanges at position 14 and 16 did not only reduce the affinity of CsaB for the acceptor, but also slightly increased the *k*_cat, acc_, indicating that a weaker binding could accelerate product dissociation.

While the R207D variant was not fully amenable to functional prediction by our models, it fully deactivated the enzyme, which indicated the essentiality of “loop 1” for enzyme function. Direct Michaelis–Menten plots for all variants upon variation of PEP and the acceptor substrate are shown in the Supplementary Figures S10–S15.

## Discussion

Given the widespread occurrence of pyruvylated compounds in nature and their manifold biological functions, it is important to increase our understanding of the enzymes which catalyze the formation of the pyruvate modification. Pyruvyltransferases transfer a pyruvate moiety from PEP to specific monosaccharide residues of diverse glycoconjugates. Notably, pyruvylation is present in various life forms but has not been found in man^1^ making it an attractive target for therapeutic intervention in the context of the rise of antibiotic resistant pathogens. Specifically pyruvylated sugars are present in a variety of bacterial cell wall glycoconjugates, such as capsular polysaccharides^25–28^, lipooligosaccharides^29^, exopolysaccharides^30,31^ and other cell wall glycopolymers^32^.

A prerequisite for a successful pyruvate transfer to occur is a suitable binding site of the PEP donor substrate on the pyruvyltransferase. PEP is mainly known as an intermediate of glycolysis and as a source of energy and phosphate^33,34^ essential to the PEP:carbohydrate phosphotransferase system (PTS) of bacteria, which catalyzes the transport and phosphorylation of numerous monosaccharides, disaccharides, amino sugars, polyols, and other sugar derivatives^35^. The PTS is organized as a four-step phosphoryl transfer system, in which phosphorylation occurs at histidyl or cysteyl residues of the phosphocarrier intermediates. These amino acid residues for PEP binding seem to be conserved also in pyruvyltransferases as evidenced by the currently investigated ketal-pyruvyltransferases CsaB from *P. alvei* and Pvg1p from *S. pombe*, where a H308 (this study) and H339^7^, respectively, were found to be PEP binders. The mechanistically best-studied pyruvyltransferase is the enol-pyruvyltransferase MurA (EC 2.5.1.7) which catalyzes the first committed step of bacterial cell wall peptidoglycan biosynthesis by transferring the enolpyruvyl part of PEP to UDP-*N*-acetylglucosamine to form UDP-*N*-acetylglucosamine enolpyruvate^36^. In the MurA mechanism, the thiolate group of a cysteine residue is pivotal to PEP binding to the enzyme. Notably, also the PEP analog fosfomycin binds to MurA; this results in enzyme inactivation making fosfomycin a potent antibiotic that is in use for cystitis^36^. Interestingly, in some bacteria, cysteine is naturally replaced by aspartic acid, retaining MurA activity but not fosfomycin susceptibility^37^. With MurA, PEP undergoes a covalent bond with cysteine or aspartic acid, preparing PEP to be transferred to UDP-*N*-acetylglucosamine and tightening the complex with UDP-*N*-acetylmuramic acid, the product of the subsequent reaction, for feedback regulation purposes^38^. Despite the fact that the enolpyruvyltransferase MurA and the ketalpyruvyltransferases CsaB and Pvg1p both use PEP as a donor substrate, MurA has only low sequence identity with the yeast enzyme Pvg1p (~ 17%) and the bacterial enzyme CsaB (19%) as well as low sequence similarity (Pvg1p: ~ 23%; CsaB: ~ 27%). Importantly, also the two ketalpyruvyltransferases have a sequence similarity of only ~ 28% /identity ~ 20%. But still, Pvg1p from *S. pombe*^7^, the only crystallized pyruvyltransferase, served as a suitable template for the homology models of CsaB generated within the frame of this study. Neither Pvg1p nor CsaB possess a conserved cysteine residue underlining a different mode of PEP binding compared to MurA.

In the study by Higuchi et al.^7^, molecular modelling based on of the partial crystal structure of Pvg1p (5ax7) revealed the amino acid residues K217 and K337 to be in direct interaction with PEP. In addition, a docking study according to Ligplot data identified H339 and L338 to interact with PEP^7^. For the key enzyme of the present study, CsaB of *P. alvei,* a combination of molecular modelling and analysis of rationally designed enzyme variants identified R148 and H308 as important PEP binding amino acid residues of CsaB (Table 1). Furthermore, K328 was identified as PEP binding residue in the docking study, supported by an enzyme activity assay (Figs. 3A, C, 4, Supplementary Fig. S10). For validation purposes, docking of PEP to 5ax7 was also done. Of note, slight differences of the binding residues in Pvg1p identified in our study compared to that from the literature^7^ can be due to a slightly differently selected docking position or due to the fact that 5ax7 was simulated^7^ (using energy minimization without a description of further details of the method used) before docking. Overall, in the present study, docking of energy optimized 5ax7 with PEP revealed R217, H339, R337 and K361 as interacting residues. The docking study of the modeled structure of CsaB indicated R148 (i.e., R217 in 5ax7), H308 (i.e., H339 in 5ax7), R306 (i.e., R337 in 5ax7). For the docking with neither of the enzymes, 5ax7 and CsaB, PEP binding to a leucine residue (L338 of 5ax7, as previously reported^7^) could be found. In contrast, for both dockings, K361 of 5ax7 and K328 of CsaB were identified to be involved in PEP binding. Furthermore, the full deactivation of a CsaB R207 variant, where R207 is located in the predicted “loop 1”, indicates the importance of the loop for enzyme activity. Notably, a comparable loop region can be predicted also for the yeast enzyme, based on our study, where R266 corresponds to R207 of CsaB (Table 1). Consequently, this study identified a flexible loop region in the CsaB model and in 5ax7 which is likely involved in catalysis.

Assaying the Y14 and F16 variants of CsaB for activity on the tailor-made mimic of their native acceptor (ManNAc-GlcNAc-PP-UndPh) underlined the proposed participation of these amino acids in acceptor (mimicked by ManNAc-GlcNAc-PP-) binding and agreed with the docking poses of the substrates (Fig. 4). This underlines the feasibility of using the acceptor devoid of its lipid appendage for modelling (ManNAc-GlcNAc-PP-) and supports the proposed SCWP biosynthesis concept where the lipid is integrated into the cytoplasmic membrane of *P. alvei* and, thus, would not be available for binding. Notably, due the use of different acceptor substrates of the bacterial and the yeast enzyme (Gal or Lac^3^ for Pvg1p versus a ManNAc-GlcNAc-PP- entity for CsaB^8^), no valid comparison of these enzymes regarding acceptor binding can be made.

This study of the CsaB enzyme from *P. alvei* provides novel insight into the structural basis of the mechanism of ketalpyruvylation of saccharides and adds to the knowledge gained from a previous study of the ortholog from *S. pombe*^7^. Despite the participation in different glycoconjugate biosynthesis pathways—prokaryotic SCWP biosynthesis versus eukaryotic *N*-glycan biosynthesis—the investigated ketalpyruvyltransferases share commonalities with regards to the binding site of the PEP donor substrate, indicative of potential similarities in the underlaying catalytic mechanism. This study provides a basis for future investigations of pyruvyltransferases as promising targets for novel anti-infective approaches. A successful example on that avenue is the use of the antibiotic fosfomycin which inactivates the MurA enzyme by targeting a cysteine residue that is essential for enzyme product release^39^. The bacterial cell wall is a well-known target for antibiotics^40^ since it is essential for protecting bacteria from the surrounding environment and maintaining their integrity. Thus, *P. alvei* CsaB whose pyruvylation activity is essential for sticking the cell wall of several Gram-positive bacteria together could be an ideal starting point for molecular docking-based virtual screening approaches to identify potential CsaB inhibitors available from databases. These could be evaluated as potential novel antibiotics.

## Methods

### Analysis of CsaB by protein structure modelling and small molecule docking for prediction of mutation sites

To obtain structural models of CsaB, three different programs/ servers were used, including I-Tasser (https://zhanglab.ccmb.med.umich.edu/I-TASSER/), Phyre2 (http://www.sbg.bio.ic.ac.uk/~phyre2/html/page.cgi?id=index) and AlphaFold (in-house installation)^41,42^. After protein structure modelling, the three modelled CsaB structures were energy-minimized to avoid modelling artefacts, *e.g.,* sidechain clashes. The modelled structures were solvated with SPC (Simple Point Charge) water, and a steepest descent minimization (maximum force < 500.0 kJ/mol/nm) using GROMACS (v2018.8) was performed. Sequence alignments were done using EMBOSS needle (https://www.ebi.ac.uk/Tools/psa/emboss_needle/) with BLOSUM80 as matrix, gap open 10, gap extend 0.5, end gap penalty false as parameters. Sequence identity and sequence similarity of the pairwise alignments was calculated after Stothard et al.^43^ (https://www.bioinformatics.org/sms2/ident_sim.html). The best template with the highest sequence identity of ~ 20% was the partial structure of the *S. pombe* pyruvyltransferase Pvg1p^7^ (pdb, 5ax7). Using 5ax7 as a template, CsaB protein structures were predicted and subsequently visualized by PyMoL (Open Source Version 2.4; https://github.com/schrodinger/pymol-open-source). Surface electrostatics for a broad pH range (4–11) were calculated using pHmap (v1.2)^22^, which automatizes the usage of ABPS (v3)^23^, pdb2pqr (v2.1.1) (https://www.poissonboltzmann.org/) and PyMol (v2.4). In short, pdb2qpr was used to predict the protonation states, ABPS was used to compute the electrostatic potential at the surface of the protein and PyMol was used to visualize the results. The electrostatic potential was visualized between a level of − 5 *RT/e* (negatively charged, red), 0 (neutral, white) to 5 *RT/e* (positively charged, blue).

Next, for comparability of data, the 5ax7 pdb structure itself was modelled using Phyre2, I-Tasser and AlphaFold and the models were used to dock PEP into the active site, which was defined based on information from the literature^7^ and the calculated surface electrostatics (this study). After defining a realistic docking pose for catalysis, considering the highest docking score and using the CAVER PyMoL plugin v3.0 and MOE with the “SiteFinder” function, respectively, to visualize the possible active site, amino acids involved in the PEP binding were predicted for, and compared between, the CsaB models. Based on this analysis, we selected the CsaB models from the AlphaFold and Phyre2 server for PEP docking, since the positions of their active site residues showed a higher similarity with the 5ax7 structure than the CsaB model from I-Tasser.

Subsequently, the minimized AlphaFold and Phyre2 CsaB model with PEP docked in the active site were used for docking the CsaB acceptor substrate^8^. The lipophilic portion of the acceptor was omitted to avoid long calculation times and to narrow down possible docking poses by keeping the docking region smaller. This approach concurs with the concept of the *P. alvei* SCWP biosynthetic pathway^8,44^. Hence, the acceptor used for docking was constituted by the β-D-ManNAc-(1 → 4)-α-D-GlcNAc-PP portion, which corresponds to the native saccharide acceptor of CsaB, containing pyrophosphate originating from its biosynthetic stage where it is linked to the lipid carrier for membrane interaction^8^.

For the docking, protein structure preparation (“Protonate3D”) and the structure building of PEP and the acceptor, MOE (Molecular Operating Environment, v2019) was used. The active/binding site for PEP and the acceptor was predicted by the “Site Finder” function and the interactions of the substrates with the active/binding site residues were evaluated by calculating the final docking scores of the ligand poses using the GBVI/WSA dG function in MOE. Of note, no solvent was present for the docking.

Based on the docking results, modelled structures and sequence alignments of CsaB and Pvg1p, sites for point mutations of CsaB were chosen aiming to target plausible PEP and acceptor binding sites. To evaluate all constructed CsaB variants, kinetic values were determined (see below).

Based on the protein structure modelling, interaction diagrams for PEP and the acceptor docked to the binding site in the CsaB AlphaFold model were calculated. Residues of the acceptor and the donor substrate within a distance of 4 Å were considered for interactions and the cut-off (minimum interaction energy required to be considered) for interaction energies for ionic and H-bond interactions was set to − 0.5 kcal/mol.

### Identification of catalytic amino acid residues in *P. alvei* CsaB by site-directed mutagenesis

Amino acids for site-directed mutagenesis were chosen based on protein structure modelling and substrate docking. Briefly, into the 1209-bp gene encoding the pyruvyltransferase CsaB from *P. alvei* CCM 2051^ T^ (PAV_RS07425), mutations were introduced to target the predicted PEP and acceptor binding sites, following an established procedure^15^. Mutations for investigating PEP binding included arginine 148 to lysine (R148K) or glutamine (R146Q), arginine 207 to aspartic acid (R207D), histidine 308 to alanine (H308A), arginine (H308R), lysine (H308K) or serine (H308S), and lysine 328 to arginine (K328R) and S88 to alanine (S88A). Mutations for investigating acceptor binding included tyrosine 14 to alanine (Y14A) or phenylalanine (Y14F), phenylalanine 16 to alanine (F16A) or arginine (F16R) and tyrosine 94 to alanine (T94A). For this purpose, overlapping forward and reverse primers, both including the desired point mutation (Supplementary Table S2) were used in separate PCR reactions in concert with plasmid pET22b-CsaB encoding C-terminally hexahistidine-tagged wild-type CsaB^8^ as a template (six cycles). Both amplicons targeting one distinct amino acid were directly mixed and amplified further in a second PCR reaction (18 cycles). Subsequently, DpnI was used to degrade methylated template-DNA and the reaction was transformed into *E. coli* DH5α for the production of the plasmids encoding in total twelve CsaB variant proteins. The purified plasmids were confirmed by DNA sequencing (Microsynth). Primers for PCR and DNA sequencing were purchased from ThermoFisher (Supplementary Table S2). For the amplification, the Phusion High-Fidelity DNA Polymerase (Fermentas) and the thermal cycler My Cycler™ (Bio-Rad) were used. The plasmids were transformed into BL21 *E. coli* for protein expression. Overexpression and purification using the Ni–NTA procedure of C-terminally hexahistidine-tagged CsaB variant proteins and CsaB wild-type, serving as a control, was done as described elsewhere^8^.

The purity of CsaB wild-type and variants was checked by 10% SDS-PAGE after Coomassie Brilliant Blue G250 staining^45^ and the protein concentration in the enzyme preparations was determined by the Bradford protein assay^46^. Subsequently, a densitometric analysis of each enzyme preparation after separation on the SDS-PA gel was done using ImageJ 1.8.0^47^ to determine the percentage of the target protein. The specific protein concentration was determined spectrophotometrically and calculated using the A_280_ extinction coefficient and molecular weight of the CsaB wild-type and mutant proteins obtained from the exPASy ProtParam tool and corrected for the densitometric value.

### Secondary structure assignment and analysis of CsaB wild-type and variants by Circular Dichroism (CD) spectroscopy

In order to assess the structural integrity of the constructed CsaB variants, their secondary structure was assigned using Database of Secondary Structure Assignments (DSSP)^48,49^ in PyMol and electronic circular dichroism (ECD) spectra in the far (180 − 260 nm)-UV regions were recorded (Chirascan, Applied Photophysics) according to Blackler et al*.*^15^, with minor modifications. Conditions were as follows: spectral bandwidth, 1 nm; scan speed, 10 s/nm; path length, 1 mm; temperature, 20 °C. The protein concentration of the recombinant, tagged proteins was measured spectrophotometrically and calculated using the A_280_ extinction coefficient and molecular weight obtained from the exPASy ProtParam too1. CsaB wild-type and variants were analyzed at a concentration of ~ 10 µM in 20 mM Tris–HCl buffer, pH 7.5. Using the Chirascan CD apparatus software the buffer background was subtracted from the protein measurement. Small concentration differences of the measurements were adjusted by overlaying the curves of wild-type and variant proteins.

### CsaB activity assay

Activity of the CsaB variants in comparison to wild-type CsaB was determined using a recently established, colorimetric phosphate release assay^9^. The assay is based on the colorimetric detection of phosphate released during pyruvyltransfer from phosphoenolpyruvate (PEP) onto the acceptor via complexation with Malachite Green and molybdate. Stock solutions of PEP and the ManNAc-GlcNAc-PP-UndPh acceptor were prepared as published previously^9^; 2, 4, 6, 10, 30, 50, 100 and 200 µM of acceptor were used for the generation of activity curves towards PEP, and 25, 50, 75, 100, 250 and 500 µM of PEP were used for measurements towards the acceptor, with each reaction containing 0.35 µg of wild-type CsaB or variant, respectively. Reactions were carried out for 5 min at 37 °C in 20 mM Tris–HCl pH 7.5, followed by color development for 1 h at 37 °C. When the PEP concentration was varied, the acceptor concentration was fixed at 150 µM due to its limited availability, while for variation of the donor, 200 µM PEP was added to the reactions. As a negative control, the enzyme was omitted from the reaction. For final analysis, the datapoints were blank-corrected and GraphPad Prism (version 9.1.2; GraphPad, San Diego, CA, USA) was used for statistical analysis, where *K*_M_ and *V*_max_ values were calculated by non-linear least-square regression to the direct Michaelis–Menten plot. Triplicate measurements were performed for all enzyme reactions.

### Supplementary Information

Supplementary Information.

## Data Availability

All data generated and analyzed during the present study are included in this published article and its supplementary information files. The corresponding original datasets are available from the corresponding author on reasonable request.
