# Supplementary material for: Molecular modelling and site-directed mutagenesis provide insight into saccharide pyruvylation by the Paenibacillus alvei CsaB enzyme
Source: Sci Rep. 2023 Aug 17;13:13394. doi: 10.1038/s41598-023-40072-1 (PMC10435577; doi:10.1038/s41598-023-40072-1)

Supplementary Information

Molecular modelling and site-directed mutagenesis provide insight into saccharide pyruvylation by the *Paenibacillus alvei* CsaB enzyme

Cordula Stefanović, Fiona F. Hager-Mair, Erik Breslmayr, Arturo López-Guzmán, Charlie Lim, Markus Blaukopf, Paul Kosma, Chris Oostenbrink, Roland Ludwig & Christina Schäffer

Supplementary Tables S1-S2

Supplementary Figures S1-S15

References

Original images (SDS-PAGE gels) for assembly of Supplementary Figure S5

Supplementary Tables

Table S1. Secondary structure assignment using DSSP in PyMol showing the Y14F, H308S and K328R CsaB variants in comparison to the CsaB wild-type enzyme (WT). H, helix; G, 3-turn helix (3_{10} helix); I, p-helix; E, extended strand; B, residue in isolated β-bridge; S, bend; T, H-bonded turn; C, coil; T, no secondary structure recognized. Differences in the predicted loop 2 (residues 180-193) of the variants compared to the WT are written in red.

**Enzyme**	**Secondary structure content**				
WT	cccccEEEEE	EEEESSccHH	HHHHHHHHHH	HHHHHHHHTT	ccEEEEEEES
K328R	cccccEEEEE	EEEESSScHH	HHHHHHHHHH	HHHHHHHHHT	ccEEEEEEES
H308S	cccccEEEEE	EEEESSScHH	HHHHHHHHHH	HHHHHHHHTT	ccEEEEEEES
Y14F	cccccEEEEE	EEEESSScHH	HHHHHHHHHH	HHHHHHHHTT	ccEEEEEEES
	1	11	21	31	41
WT	SHHHHHHHHS	SEEEETTcHH	HHHHHHHHcS	EEEEcSEEEE	cSSScTTHHH
K328R	SHHHHHHHHS	SEEEETTcHH	HHHHHHHHcS	EEEEcSEEEE	cSSScTTHHH
H308S	SHHHHHHHHS	SEEEETTcHH	HHHHHHHHcS	EEEEcSEEEE	cSSScTTHHH
Y14F	SHHHHHHHHS	SEEEETTcHH	HHHHHHHHcS	EEEEESSScB	cSSScTTHHH
	51	61	71	81	91
WT	HHHHHHHHHH	HTTccEEEEE	EEEccccccGG	GHHHHHHHHT	TSSEEEEESSH
K328R	HHHHHHHHHH	HTTccEEEEE	EEEccccccGG	GHHHHHHHHT	TcSEEEEESSH
H308S	HHHHHHHHHH	HTTccEEEEE	EEEccccccGG	GHHHHHHHHT	TcSEEEEESSH
Y14F	HHHHHHHHHH	HTTccEEEEE	EcBccccccGG	GHHHHHHHHT	TSSEEEEESSH
	101	111	121	131	141
WT	HHHHHHHHTT	ccGGGcEEcc	cGGGGccccT	TTScccccccS	STTBcTTScB
K328R	HHHHHHHHTT	ccGGGcEEcc	cGGGGccccS	STTSccSScS	SSSBcTTScB
H308S	HHHHHHHHTT	ccGGGcEEcc	cGGGGccccH	HHHcccTTcT	TTTBcTTScB
Y14F	HHHHHHHHTT	ccGGGcEEcc	cGGGGccccT	TSccccccccS	STTBcTTScB
	151	161	171	181	191
WT	EEEEEccccS	TTcHHHHHHH	HHHHHHHHHS	ccEEEEccSS	HHHHHHHHHH
K328R	EEEEEccccS	TTcHHHHHHH	HHHHHHHHHS	ccEEEEccSS	HHHHHHHHHH
H308S	EEEEEccccS	TTcHHHHHHH	HHHHHHHHHS	ccEEEEccSS	HHHHHHHHHH
Y14F	EEEEEccccS	TTcHHHHHHH	HHHHHHHHHS	ccEEEEccSS	HHHHHHHHHH
	201	211	221	231	241
WT	HHHHHTTTSc	BcTTSScccc	ccTTScccEE	EEccccSSHH	HHHHHHHTcS
K328R	HHHHHHHHSc	TTTSccBSSc	TTccSSScEE	EEccccSSHH	HHHHHHHTcS
H308S	HHHHHTTTSc	BcTTSSSccc	ccccSSScEE	EEccccSSHH	HHHHHHHTcS
Y14F	HHHHHTTTSc	BcTTSSSccc	ccccSSScEE	EEccccSSHH	HHHHHHHTcS
	251	261	271	281	291

WT	EEEESSHHHH HHHHHTTccE EEEESSHHHH HHHHHTTccc SEETTBccHH
K328R	EEEESSHHHH HHHHHTTccE EEEESSHHHH HHHHHTTccc SEETTBccHH
H308S	EEEESSHHHH HHHHHTTccE EEEESSHHHH HHHHHTTccc SEETTBccHH
Y14F	EEEESSHHHH HHHHHTTccE EEEESSHHHH HHHHHTTccc SEETTBccHH
	301 311 321 331 341
WT	HHHHHHHHHH HTHHHHHHHH HHHHHHHHHH HHHHHHHHHH HHHHHc
K328R	HHHHHHHHHH HTHHHHHHHH HHHHHHHHHH HHHHHHHHHH HHHHHc
H308S	HHHHHHHHHH HTHHHHHHHH HHHHHHHHHH HHHHHHHHHH HHHHHc
Y14F	HHHHHHHHHH HTHHHHHHHH HHHHHHHHHH HHHHHHHHHH HHHHHc
	351 361 371 381 391

Table S2. Oligonucleotide primers used in this study. Mutated base triplets are colored in red.

**Primer**	**Sequence**
Y14F_fwd1.8	GTA CTT TCC GGA TAT TTC GGA TTC AAT AAT AGT
Y14F_rev1.8	ACT ATT ATT GAA TCC GAA ATA TCC GGA AAG TAC
F16R_fwd0.2	TCC GGA TAT TAC GGA AGA AAT AAT AGT GGT GAC
F16R_rev0.2	GTC ACC ACT ATT ATT TCT TCC GTA ATA TCC GGA
F16A_fwd2.1	TCC GGA TAT TAC GGA GCA AAT AAT AGT GGT GAC
F16A_rev2.1	GTC ACC ACT ATT ATT CGT TCC GTA ATA TCC GGA
Fwd-R148Q-1.3	GCGTATGTTTCGGTACAAGATCGTGAGTCTGCA
rev-R148Q-1.3	TGCAGACTCACGATCTTGTACCGAAACATA
Fwd-R148Q-2.9	GCGTATGTTTCGGTACAGGATCGTGAGTCTGCA
rev-R148Q-2.9	TGCAGACTCACGATCCTGTACCGAAACATA
For-R148K-3.8	GCGTATGTTTCGGTAAAAGATCGTGAGTCTGCA
rev-R148K-3.8	TGCAGACTCACGATCCTTTACCGAAACATACGC
fwd-R207D-3.3	GGCGTGTCCCTCGATTTTTGGAATCAG
rev-207D-3.3	CTGATTCCAAAAATCGAGGGACACGCC
fwd-207D-2.3	GGCGTGTCCCTCGACTTTTGGAATCAG
rev-207D-2.3	CTGATTCCAAAAGTCGAGGGACACGCC
Fwd-H308A-2.1	GTTGGAATGCGATTGGCATCCCTCATTTATGCG
rev-H308A-2.1	CGCATAAATGAGGGATGCCAATCGCATTCCAAC
fwd-H308A-2.3	GTTGGAATGCGATTGGCCTCCCTCATTTATGCG
rev-H308A-2.3	CGCATAAATGAGGGAGGCCAATCGCATTCCAAC
For-H308R-2.2	GTTGGAATGCGATTGCGCTCCCTCATTTATGCG
Rev-H308R-2.2	CGCATAAATGAGGGAGCGCAATCGCATTCCAAC
For-H308R-2.4	GTTGGAATGCGATTGCGTTCCCTCATTTATGCG
Rev-H308R-2.4	CGCATAAATGAGGGAACGCAATCGCATTCCAAC
H308K_fwd1.2	GTT GGA ATG CGA TTG AAG TCC CTC ATT TAT GCG
H308K_rev1.2	CGC ATA AAT GAG GGA CTT CAA TCG CAT TCC AAC
H308S_fwd	GTT GGA ATG CGA TTG AGC TCC CTC ATT TAT GCG
H308S_rev	CGC ATA AAT GAG GGA TCG CAA TCG CAT TCC AAC
K328R_fwd0.2	ATT TCT TAT GAT CCG AGA ATT GAT CAG TTT TTG
K328R_rev0.2	CAA AAA CTG ATC AAT TCT CGG ATC ATA AGA AAT

Supplementary Figures

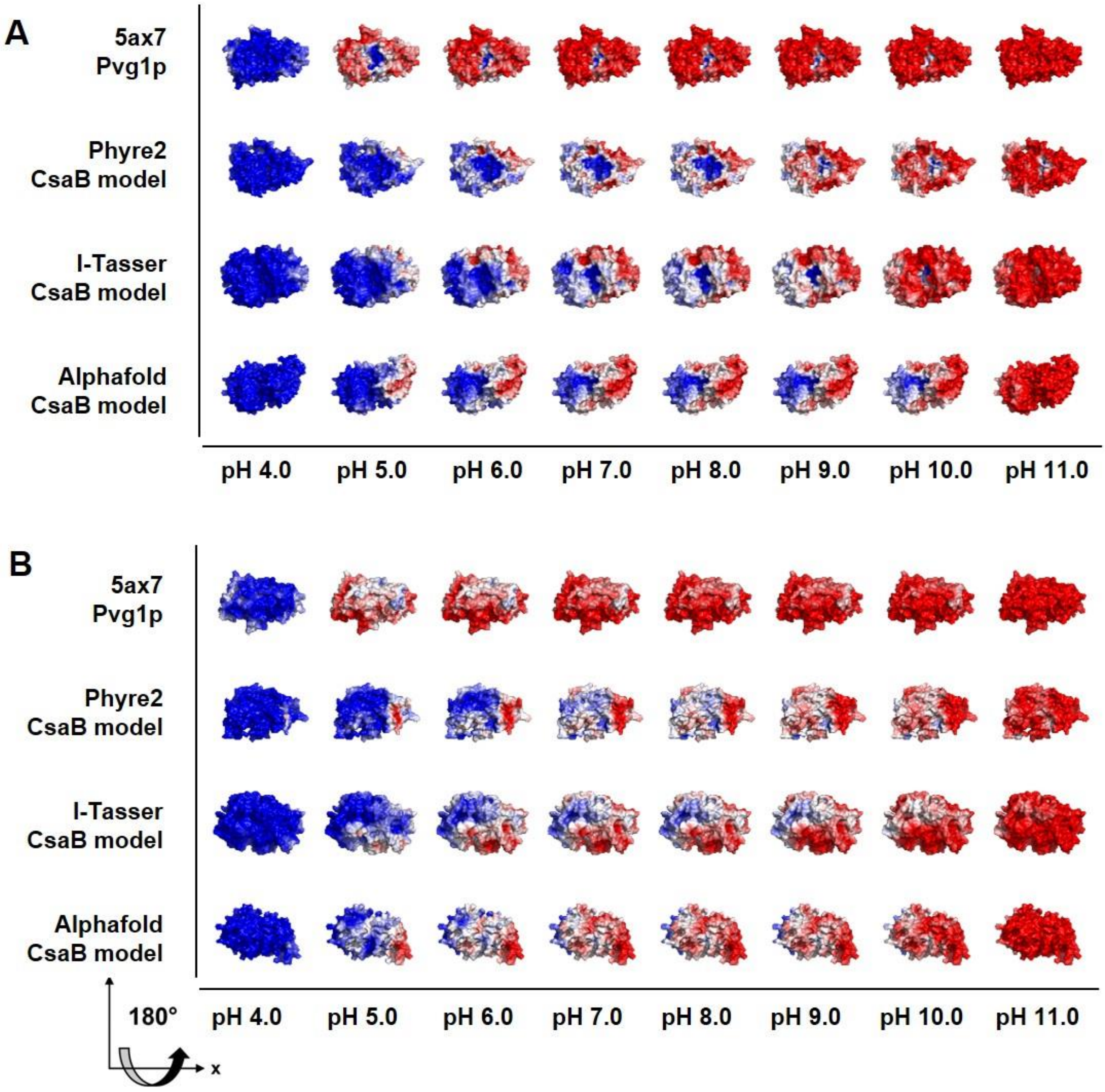

Figure S1. Surface electrostatics at pH 4, 5, 6, 7, 8, 9, 10, and 11 of Pvg1p from *S. pombe* (5ax7) in comparison to the models of CsaB from *P. alvei* (**A**); representations turned by180° (**B**). Surface areas with a positive electrostatic potential (+5 *RT/e*) are colored in blue, surface areas with a negative electrostatic potential (-5 *RT/e*) in red. Surface electrostatics were calculated using pHmap (v1.2)[1], which automatizes the usage of ABPS (v3)[2], pdb2pqr (v2.1.1) (https://www.poissonboltzmann.org/) and PyMol (v2.4).

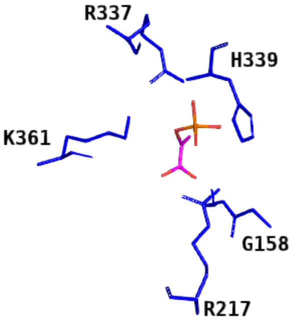

Figure S2. Zoomed-in view of the 5ax7 crystal structure of Pvg1p (blue) with docked PEP (magenta) to show PEP binding amino acids of Pvg1p, based on Higuchi *et al.*[3] (*i.e.*, R337 and R217 in Pvg1p) and on a docking study in analogy to the situation found in CsaB (this study; *i.e.*, R217, H339, K361. R337 and G159 in Pvg1p). The 5ax7 protein structure with docked substrate was visualized by PyMoL (Open Source Version 2.4; https://github.com/schrodinger/pymol-open-source).

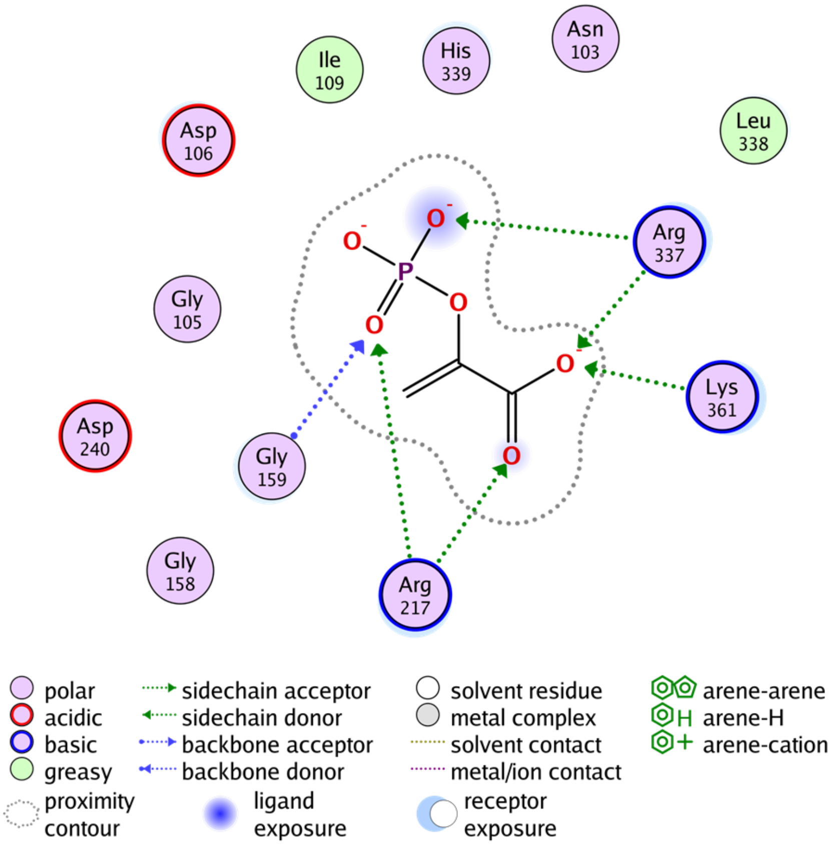

Figure S3. Ligand interaction diagram for the PEP donor docked to the binding site in the 5ax7 protein structure.

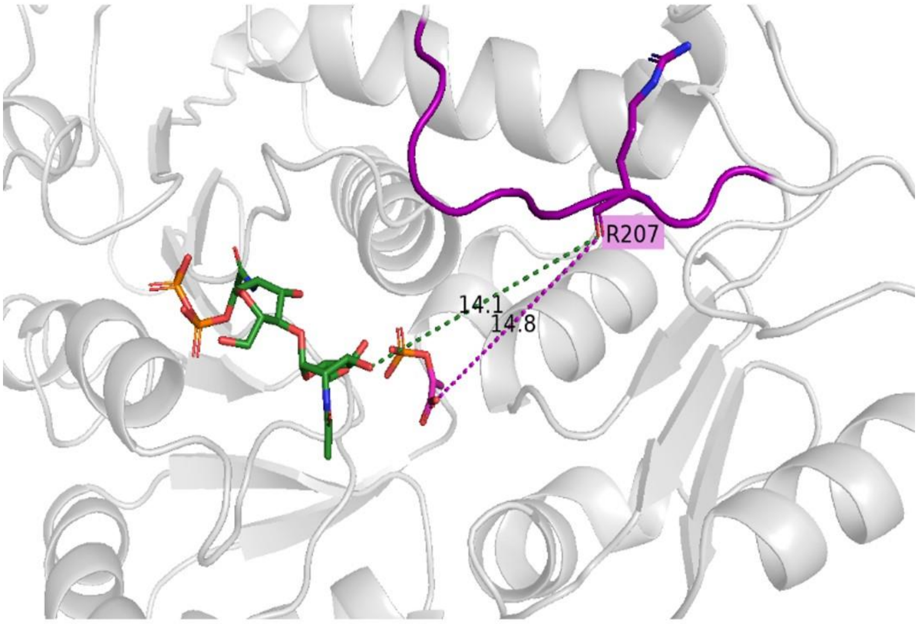

Figure S4. Enlarged view of "loop 1" (purple) in CsaB modelled by Phyre2 with docked substrates. The closest atom of PEP (magenta) to the conserved arginine residue R207 is at 14.8-Å distance (dotted line in magenta), the closest atom of the acceptor (green with phosphates in orange) to R207 is at 14.1-Å distance (dotted line in green). The CsaB protein structure with docked substrate was visualized by PyMoL (Open Source Version 2.4; https://github.com/schrodinger/pymol-open-source).

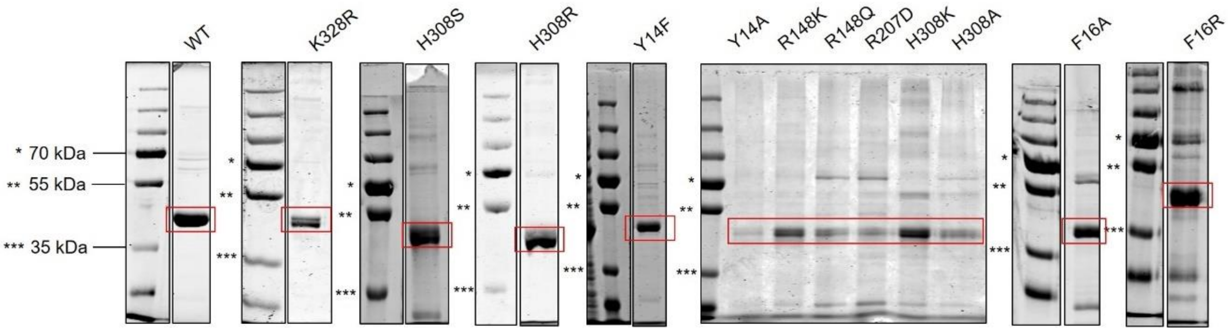

Figure S5. SDS-PAGE 10% gel of CsaB wild-type and variants upon Coomassie Brilliant Blue G250 staining after Ni-NTA purification. All recombinant enzymes (indicated in a red frame) are accompanied by a MW marker run on the same gel. Standard, PageRuler Prestained Plus (left). *, 70 kDa, **, 55 kDa, ***, 35 kDa. The original gels, from which the lanes were cropped are shown at the end of the Supplementary Information file.

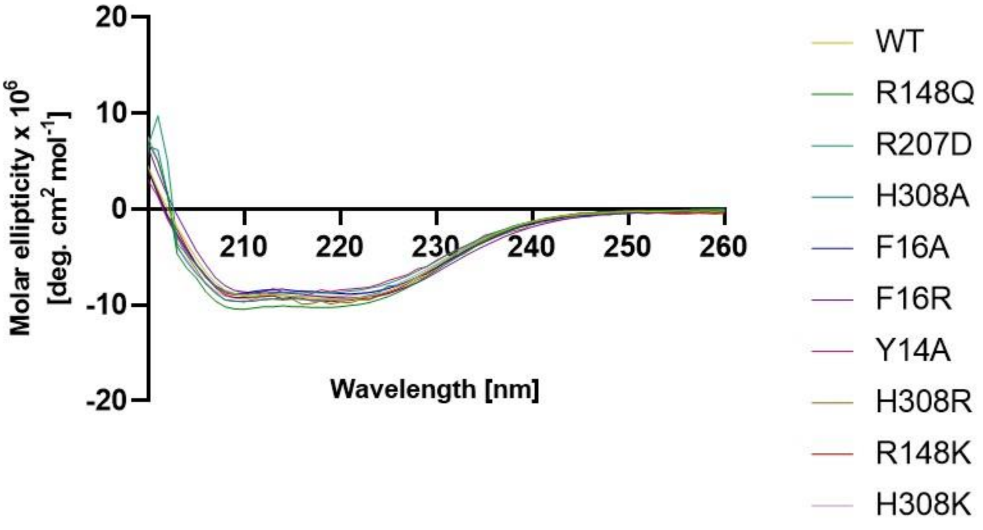

Figure S6. Far-UV-ECD spectroscopy of wild-type CsaB and the R148Q, R207D, H308A, K328R, F16A, F16R, Y14A, H308R, and R148K CsaB variants, showing identical curve shapes indicative of retainment of the native secondary structure in the variants.

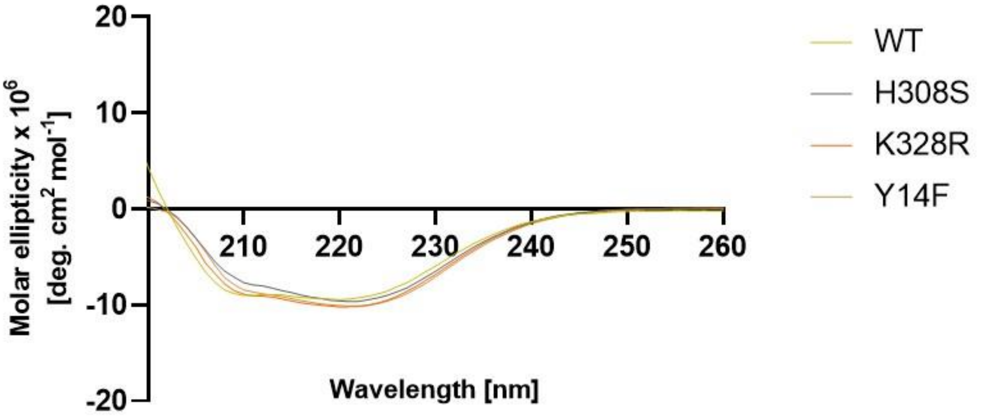

Figure S7. Far-UV-ECD spectroscopy of the Y14F, H308S and K328R variants compared to wild-type CsaB, showing a slightly different curve shape of the variants putatively pinpointing differences in the secondary structure.

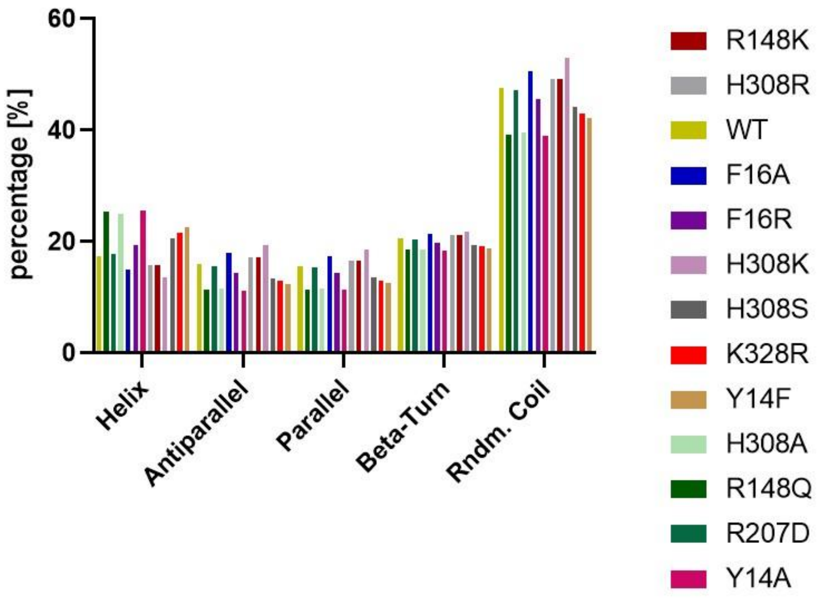

Figure S8. Calculated percentages of secondary structure elements of CsaB wild-type and CsaB variants using CDNN between 210-280nm. CDNN was calculated using the Chirascan software.

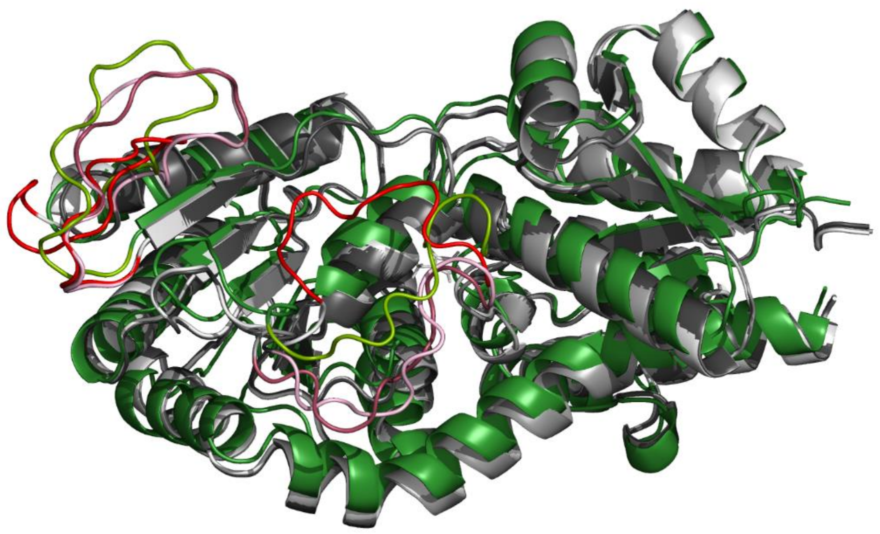

Figure S9. Overlay of AlphaFold models of the CsaB variants Y14F (gray 30), H308S (gray 60) and K328R (gray 90) showing differences in loop regions between residues 180-193 (“loop 2”; in the centre of the proteins) and residues 260-278, compared to wild-type CsaB (green). The loop regions of Y14F, H308S, K328R and wild-type CsaB are shown in light pink, raspberry, red and splitpea, respectively. The confidentiality of the loop regions is below 90 pLDDT. (Note that upon colouring of the loop regions, the confidentiality colouring of the AlphaFold models is removed.) The CsaB protein structure was visualized by PyMoL (Open Source Version 2.4; https://github.com/schrodinger/pymol-open-source).

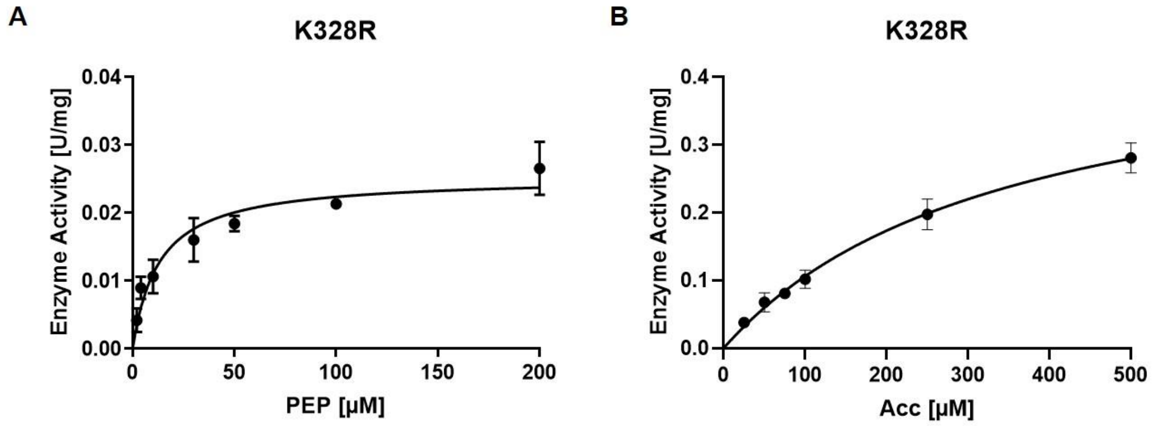

Figure S10. Kinetic analysis of K328R CsaB variant activity revealing K_{M} and k_{cat}. Direct Michaelis–Menten plot for varying PEP concentration (**A**) and upon variation of the acceptor (**B**). GraphPad Prism (version 9.1.2; GraphPad, San Diego, CA, USA) was used for statistical analysis, where K_{M} and V_{max} values were calculated by non-linear least-square regression to the direct Michaelis–Menten plot.

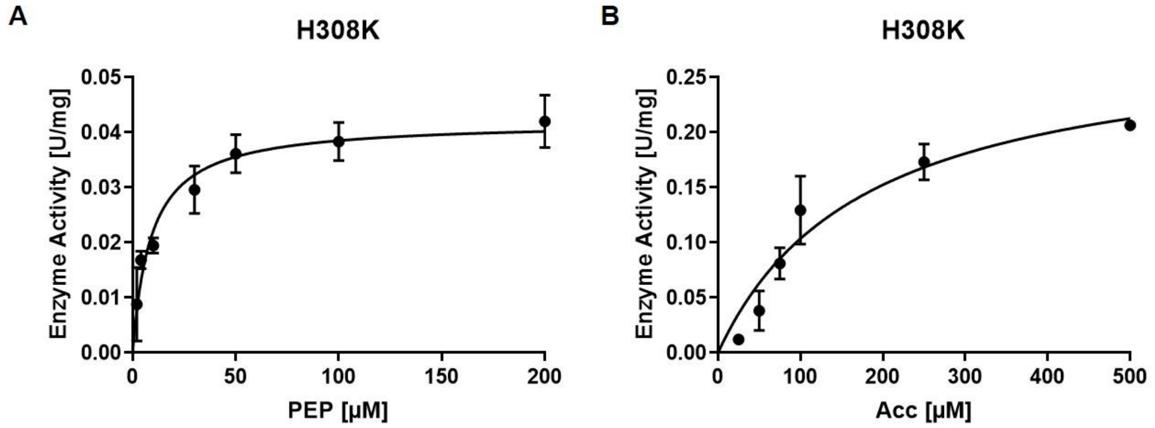

Figure S11. Kinetic analysis of H308K CsaB variant activity revealing K_{M} and k_{cat}. Direct Michaelis–Menten plot for varying PEP concentration (**A**) and upon variation of the acceptor (**B**). GraphPad Prism (version 9.1.2; GraphPad, San Diego, CA, USA) was used for statistical analysis, where K_{M} and V_{max} values were calculated by non-linear least-square regression to the direct Michaelis–Menten plot.

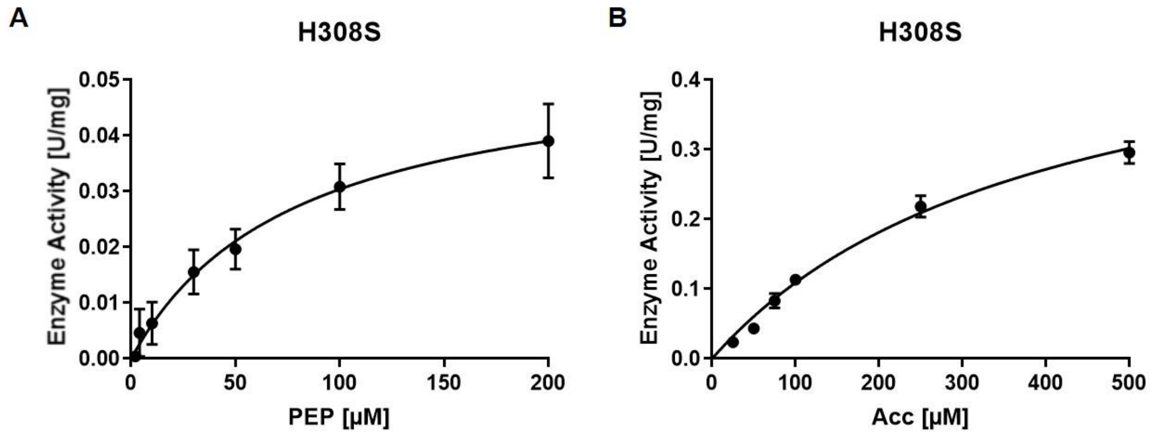

Figure S12. Kinetic analysis of H308S CsaB variant activity revealing K_M and k_{cat}. Direct Michaelis–Menten plot for varying PEP concentration (**A**) and upon variation of the acceptor (**B**). GraphPad Prism (version 9.1.2; GraphPad, San Diego, CA, USA) was used for statistical analysis, where K_M and V_{max} values were calculated by non-linear least-square regression to the direct Michaelis–Menten plot.

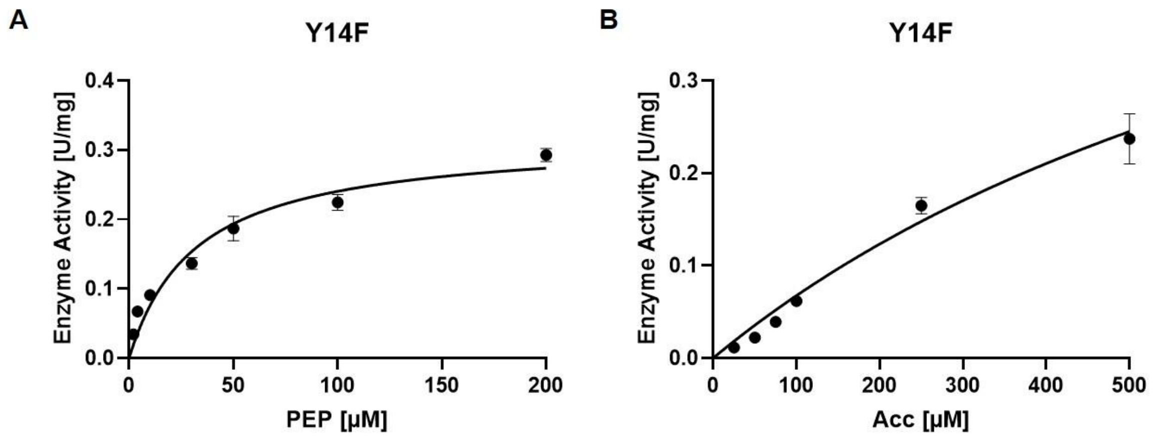

Figure S13. Kinetic analysis of Y14F CsaB variant activity revealing K_M and k_{cat}. Direct Michaelis–Menten plot for varying PEP concentration (**A**) and upon variation of the acceptor (**B**). GraphPad Prism (version 9.1.2; GraphPad, San Diego, CA, USA) was used for statistical analysis, where K_M and V_{max} values were calculated by non-linear least-square regression to the direct Michaelis–Menten plot.

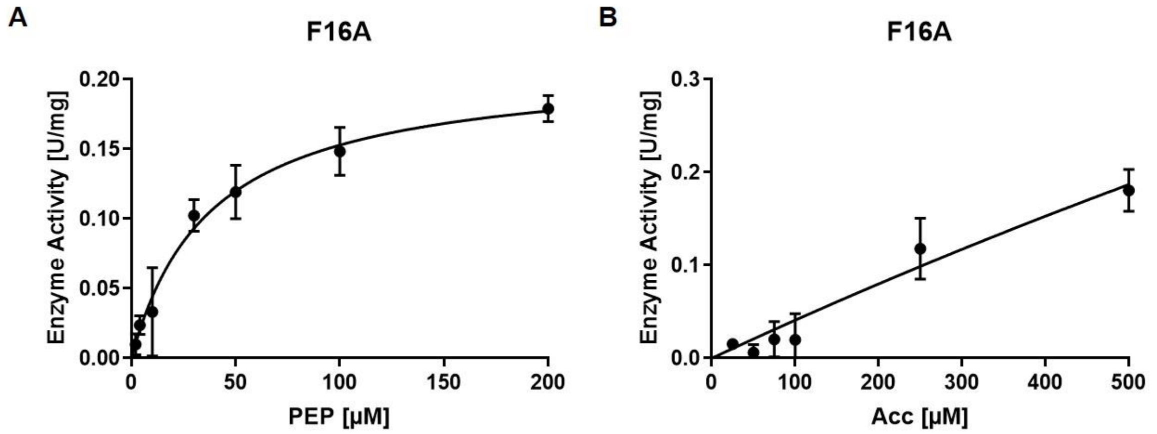

Figure S14 Kinetic analysis of F16A CsaB variant activity revealing K_M and k_{cat}. Direct Michaelis–Menten plot for varying PEP concentration (**A**) and upon variation of the acceptor (**B**). GraphPad Prism (version 9.1.2; GraphPad, San Diego, CA, USA) was used for statistical analysis, where K_M and V_{max} values were calculated by non-linear least-square regression to the direct Michaelis–Menten plot.

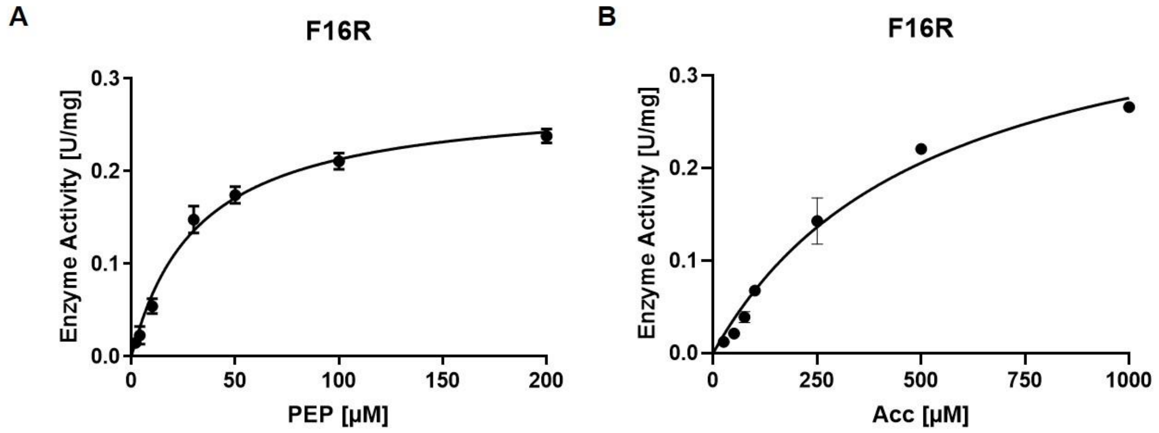

Figure S15. Kinetic analysis of F16R CsaB variant activity revealing K_M and k_{cat}. Direct Michaelis–Menten plot for varying PEP concentration (**A**) and upon variation of the acceptor (**B**). GraphPad Prism (version 9.1.2; GraphPad, San Diego, CA, USA) was used for statistical analysis, where K_M and V_{max} values were calculated by non-linear least-square regression to the direct Michaelis–Menten plot.

References

1 Breslmayr, E. pHmap - A tool for automatized calculation and visualization of protein surface charge pH-profiles. *Zenodo* **v1.2**, doi:10.5281/zenodo.4751499 (2021).

2 Jurrus, E. *et al.* Improvements to the APBS biomolecular solvation software suite. *Protein Sci.* **27**, 112-128, doi:10.1002/pro.3280 (2018).

3 Higuchi, Y. *et al.* A rationally engineered yeast pyruvyltransferase Pvg1p introduces sialylation-like properties in neo-human-type complex oligosaccharide. *Sci. Rep.* **6**, 26349, doi:10.1038/srep26349 (2016).

Original images (SDS-PAGE gels) for assembly of Supplementary Figure S5

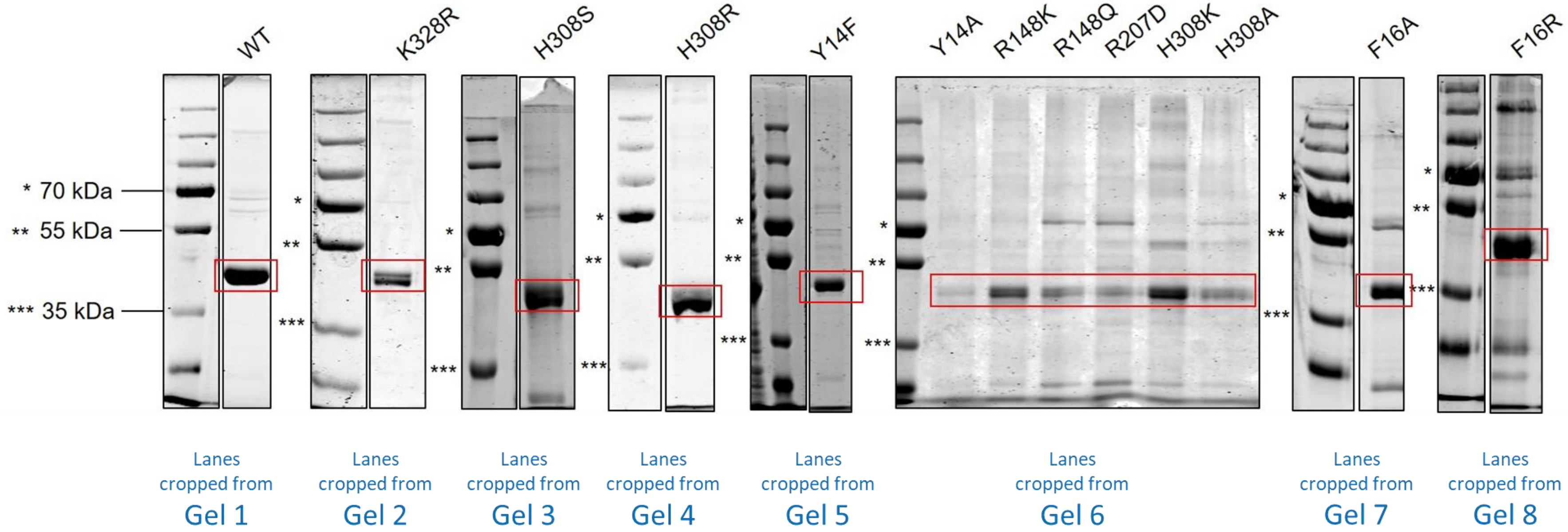

Cropping is detailed for each Coomassie-stained SDS-PAGE gel on the next pages.
Lanes cropped in the gels are shown inside black, broken frames.

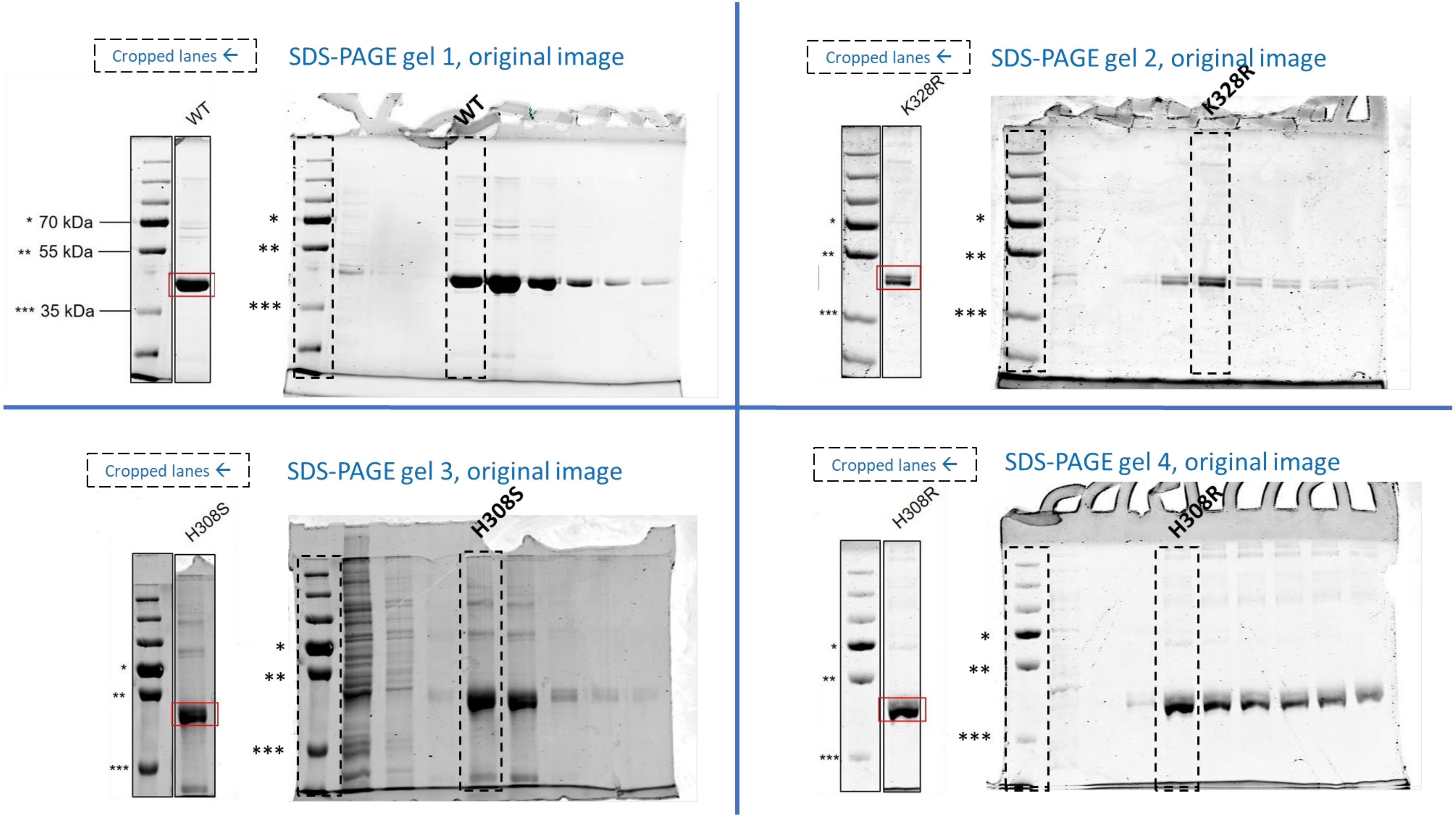
Cropped lanes
SDS-PAGE gel 1, original image
WT
* 70 kDa
** 55 kDa
*** 35 kDa
*
**
***
WT
Cropped lanes
SDS-PAGE gel 2, original image
K328R
*
**
***
*
**
***
K328R
Cropped lanes
SDS-PAGE gel 3, original image
H308S
*
**
***
*
**
***
H308S
Cropped lanes
SDS-PAGE gel 4, original image
H308R
*
**
***
*
**
***
H308R

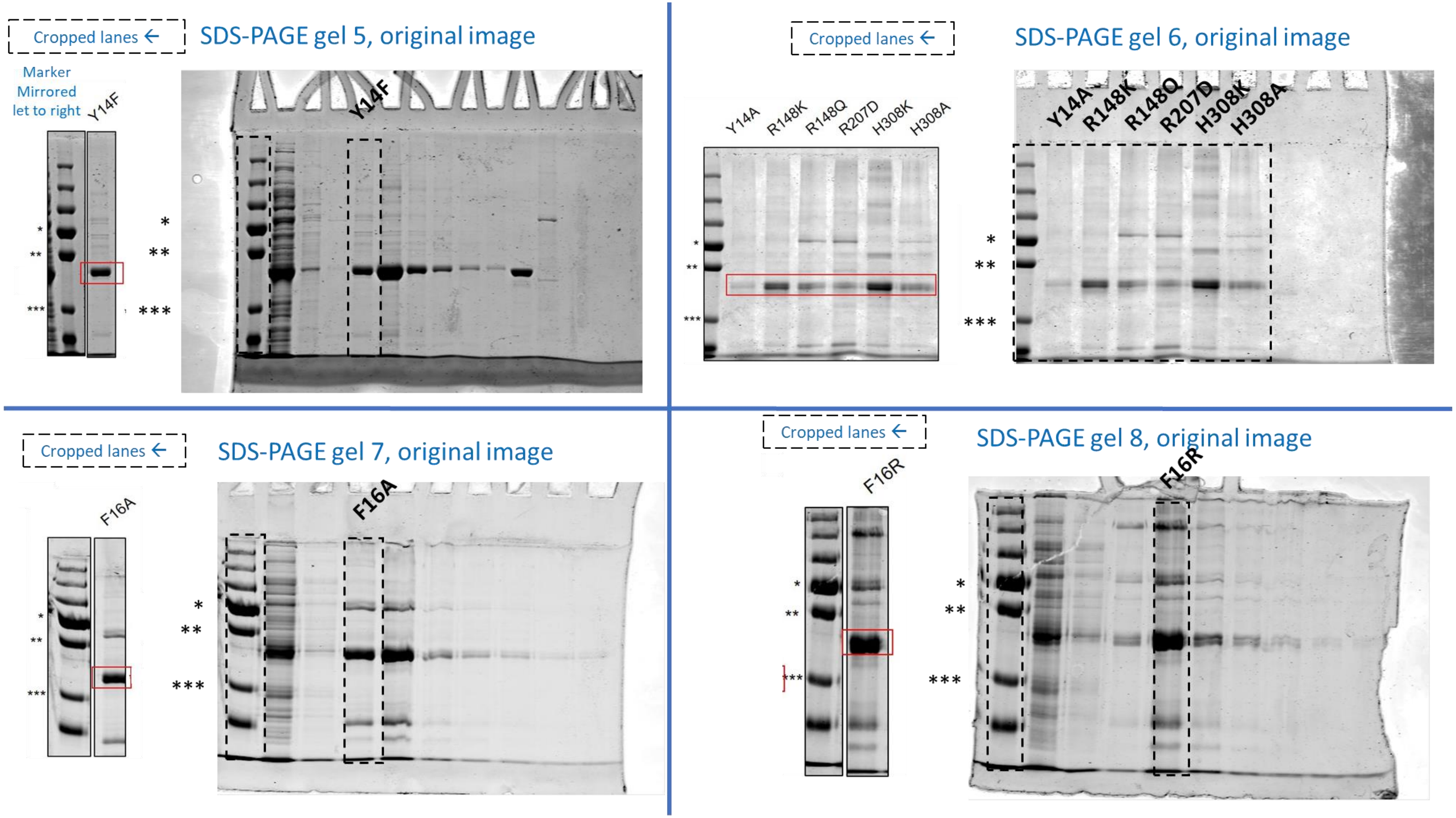

Cropped lanes ←
SDS-PAGE gel 5, original image
Marker
Mirrored
let to right
Y14F
*
**
***
Y14F
Cropped lanes ←
SDS-PAGE gel 6, original image
Y14A
R148K
R148Q
R207D
H308K
H308A
*
**
***
Y14A
R148K
R148Q
R207D
H308K
H308A
Cropped lanes ←
SDS-PAGE gel 7, original image
F16A
*
**
***
F16A
Cropped lanes ←
SDS-PAGE gel 8, original image
F16R
*
**
***
F16R

SDS-PAGE gels without explanations

Gel 1

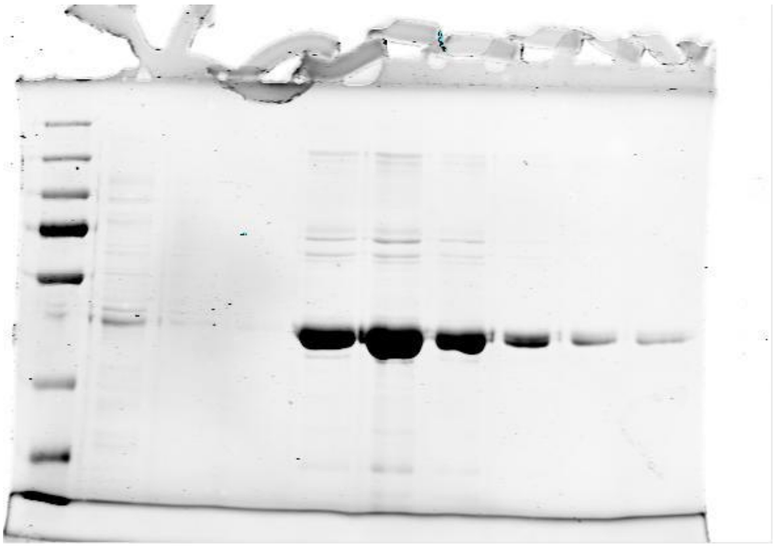

Gel 2

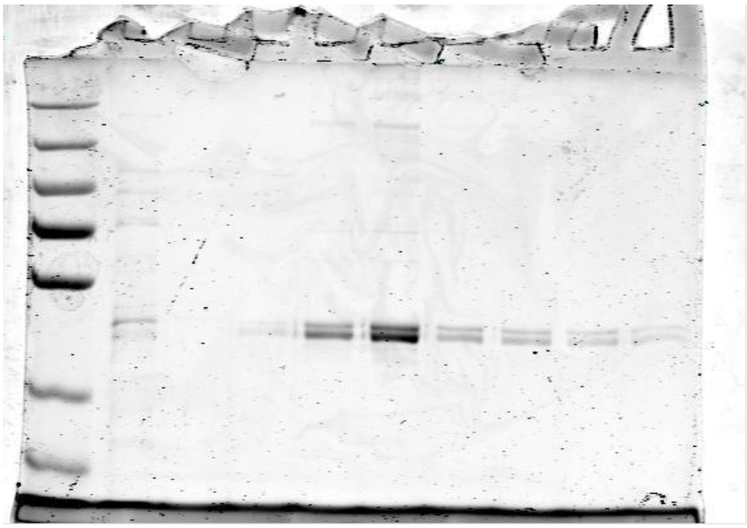

Gel 3

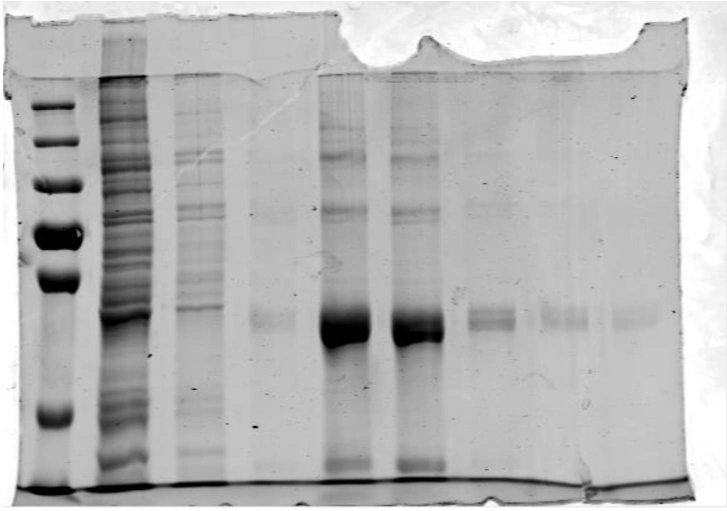

Gel 4

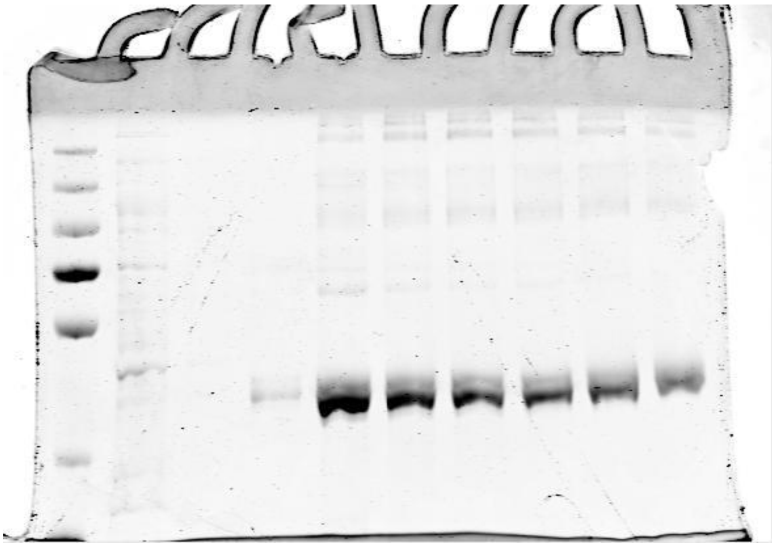

Gel 5

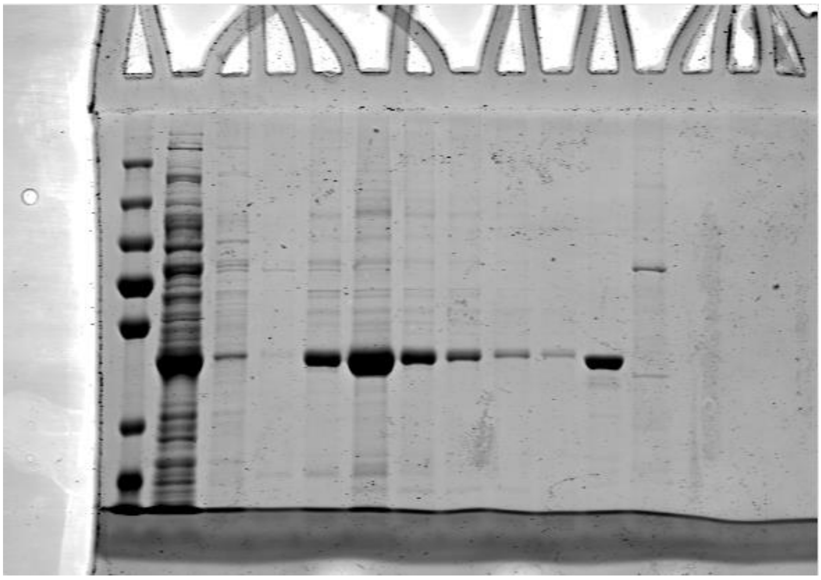

Gel 6

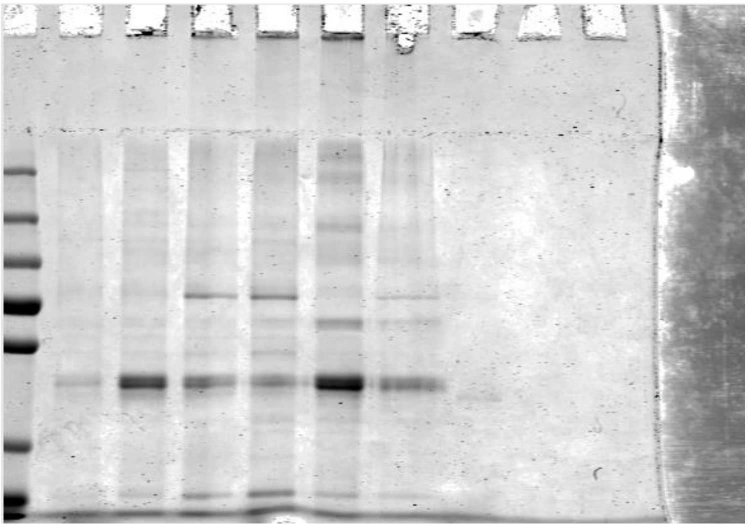

Gel 7

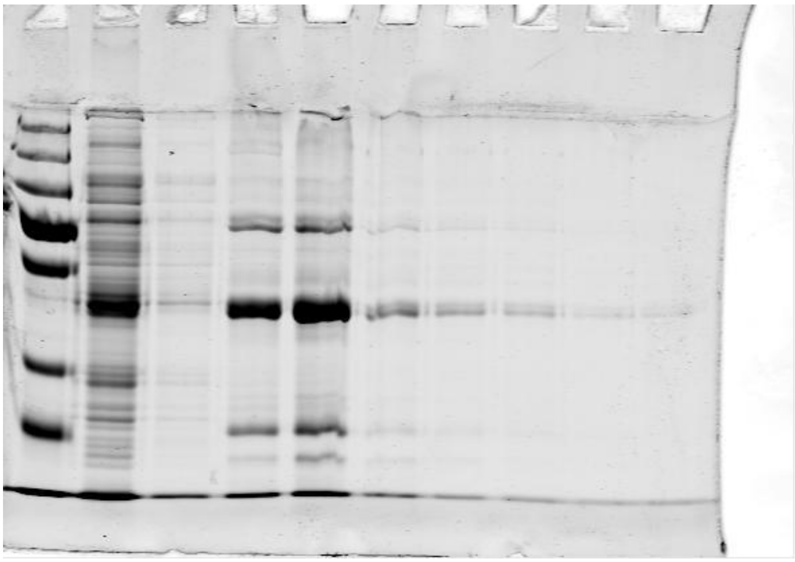

Gel 8

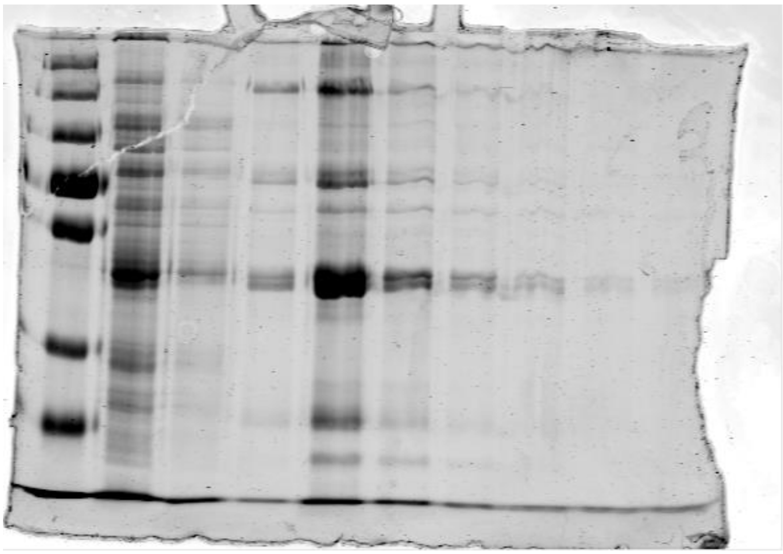

Supplement: Supplementary file 1 — Supplementary Information. [file 41598_2023_40072_MOESM1_ESM.pdf]
